# Soluble adenylyl cyclase in nonmammalian sperm is directly controlled by pH, not by HCO_3_^−^ or Ca^2+^

**DOI:** 10.1073/pnas.2505026123

**Published:** 2026-01-27

**Authors:** Olivia Kendall, Oanh Tu Hoang, Joshua L. Wort, Hussein Hamzeh, Heinz G. Körschen, René Pascal, Kai Korsching, Meritxell Wu-Lu, Wolfgang Bönigk, Christian Kambach, Luis Alvarez, Reinhard Seifert, Timo Strünker, Maria Andrea Mroginski, U. Benjamin Kaupp

**Affiliations:** ^a^Max Planck Institute for Neurobiology of Behavior–caesar, Molecular Sensory Systems Group, Bonn 53175, Germany; ^b^Life and Medical Sciences Institute, Molecular Sensory Systems, University of Bonn, Bonn 53115, Germany; ^c^Department of Chemistry, Faculty II, Technical University Berlin, Berlin 10623, Germany; ^d^Marine Biological Laboratory, Woods Hole, MA 02543; ^e^Department of Biochemistry, University of Bayreuth, Bayreuth 95440, Germany; ^f^Max Planck Institute for Neurobiology of Behavior–caesar, Neural Information Flow Group, Bonn 53175, Germany; ^g^Center of Reproductive Medicine and Andrology, Molecular Reproductive Physiology, University Hospital Münster, University of Münster, Münster 48149, Germany; ^h^Max-Planck-Institute for Multidisciplinary Sciences, Biophysics of Cellular Signal Transduction, Göttingen 37077, Germany

**Keywords:** soluble adenylyl cyclase (sAC), cAMP, sperm, fertilization, pH

## Abstract

In mammalian sperm, high HCO_3_^−^ concentrations in semen and the oviduct activate soluble adenylyl cyclase (sAC), which synthesizes cyclic AMP to regulate sperm motility. Here, we demonstrate that sAC orthologs in echinoderms and fish, species whose sperm fertilize eggs in aquatic environments with low HCO_3_^−^, are activated by alkaline pH rather than HCO_3_^−^. In human sAC, two charged residues coordinate HCO_3_^−^ and are essential for HCO_3_^−^-mediated activation. In contrast, pH-regulated sACs and orthologs from 13 phyla have these residues replaced by neutral ones. These substitutions abolish the enzyme’s responsiveness to HCO_3_^−^ and suggest that pH regulation of sAC is widespread in nonmammalian metazoans. Furthermore, we show that in sea urchin sperm, a rise of pH during spawning stimulates cAMP synthesis, a key step in the activation of motility. An evolutionarily significant pattern is emerging: Across phyla, sAC regulation by pH or HCO_3_^−^ represents an adaptation to environments with low or high HCO_3_^−^, respectively.

## Significance

Cyclic AMP (cAMP) is a cellular messenger vital for key sperm functions, including motility and fertilization capacity. The enzyme soluble adenylyl cyclase (sAC) synthesizes cAMP. While it was thought that bicarbonate regulation of sAC was conserved across species, from corals to humans, this does not apply to nonmammalian metazoans. In mammals, bicarbonate activates sAC, whereas in marine invertebrates and fish, sAC is unresponsive to bicarbonate but is instead activated by alkaline pH, functioning as a direct pH sensor. These findings help build a comprehensive model of cAMP signaling in aquatic animal sperm. The pH regulation of sAC in aquatic animals makes their reproductive success vulnerable to increasing ocean acidification driven by climate change.

Cyclic AMP (cAMP) is a widespread cellular messenger that governs downstream targets in numerous signaling pathways. In most metazoans, cAMP is synthesized by two kinds of adenylyl cyclases (ACs): transmembrane ACs (tmAC), which are regulated by G proteins (1, 2), and a soluble AC (sAC), which is regulated by HCO_3_^−^ and Ca^2+^ but not G-proteins (3, 4). sAC was first discovered in mammalian sperm as a unique AC due to its notably high sensitivity to manganese (Mn^2+^) compared to magnesium (Mg^2+^) (5, 6). Later, its atypical regulation by bicarbonate (HCO_3_^−^) and calcium (Ca^2+^) was also recognized (3, 7).

The concentrations of HCO_3_^−^ and pH are inextricably linked through the chemical equilibrium:

$${\mathrm{CO}}_{2}+H_{2}O⇌{\mathrm{HCO}}_{3}^{-}+H^{+}.$$

Because of this relationship, sAC can translate changes in this equilibrium–specifically changes in intracellular pH (pH_i_) that alter HCO_3_^−^ levels–into a cellular cAMP signal. This led to the proposal that sAC indirectly functions as a “general cellular pH sensor” (4, 8–10). At the same time, it was emphasized that sAC itself is not directly affected by pH (3, 11).

The critical roles of HCO_3_^−^-stimulated cAMP production by sAC are well established in mammals (for review, see ref. 4), including human sperm (12, 13). The high HCO_3_^−^ concentrations (15 to 25 mM) found in semen (14) and the oviduct (15) trigger cAMP synthesis. This, in turn, initiates sperm motility and capacitation, a maturation process essential for mammalian sperm to be able to fertilize an egg (16). Considering that tmACs are neither functionally detected (17) nor found at the protein level in human sperm (18) and that cAMP levels are virtually undetectable in highly purified sperm from mice lacking sAC (sAC^−/−^) (19), it is likely that sAC is the exclusive source of cAMP synthesis that contributes to motility (see also ref. 20).

Crystal structures of human sAC reveal that HCO_3_^−^ is bound via two positively charged residues (K95 and R176) (21, 22). Substituting these residues with neutral ones substantially reduces cAMP synthesis (21). It has been reported that these critical residues and the catalytic domain are highly conserved (23) and that in sperm of marine invertebrates and fish, sAC is also activated by HCO_3_^−^ (see *SI Appendix*, Table S1 for references and discussion). Thus, the prevailing view has been that sAC is regulated by HCO_3_^−^ and Ca^2+^ across different species (4).

For external fertilizers in marine habitats, cAMP is associated with activation of sperm motility during spawning (24–26) and is involved in chemotactic navigation (27–29). However, sAC activation by HCO_3_^−^ in marine species presents a paradox. Unlike mammalian sperm, which experience an increase of HCO_3_^−^ from ca. 4 mM in the testis to ca. 25 mM in semen (14) or oviduct (15), marine habitats have low partial CO_2_ pressure (pCO_2_), which results in low aqueous and intracellular HCO_3_^−^ concentrations in marine invertebrates and fish (only 1 to 5 mM) (11). Given a Michaelis–Menten constant (K_M_) of 14 to 28 mM for human sAC (3, 21, 30), a HCO_3_^−^-sensitive sAC would be poorly activated under these conditions. Therefore, sperm are not exposed to a significant increase of HCO_3_^−^ during spawning or chemotactic navigation to the egg. These considerations are relevant to many species beyond echinoderms, as variants of this signaling pathway exist in sperm of other marine invertebrates and fish (31–34).

This study resolves the long-standing puzzle of how spawning or chemoattractants activate sAC and increase cAMP levels in sperm of sea urchin and fish. We show that sAC isoforms from *Arbacia punctulata* and *Salmo salar* are activated by alkaline pH, not by HCO_3_^−^ or Ca^2+^. The residues that bind HCO_3_^−^ in mammalian sAC are not conserved in *A. punctulata* and *S. salar.* With few exceptions, these residues are also not conserved in nonmammalian species from 13 phyla, suggesting that direct pH control of sAC is common among metazoans. The chemotactic guidance of sea urchin sperm depends on pH-controlled sAC. We conclude that regulation of sAC has evolved to respond to key internal signals in sperm, such as rising HCO_3_^−^ levels in mammalian sperm or alkalization in fish and marine invertebrates. Ongoing ocean acidification could interfere with sAC activity, potentially harming external reproduction in marine environments.

## Results

### Mammalian and Nonmammalian sAC Differ in Two Key Residues.

**Fig. 1.**
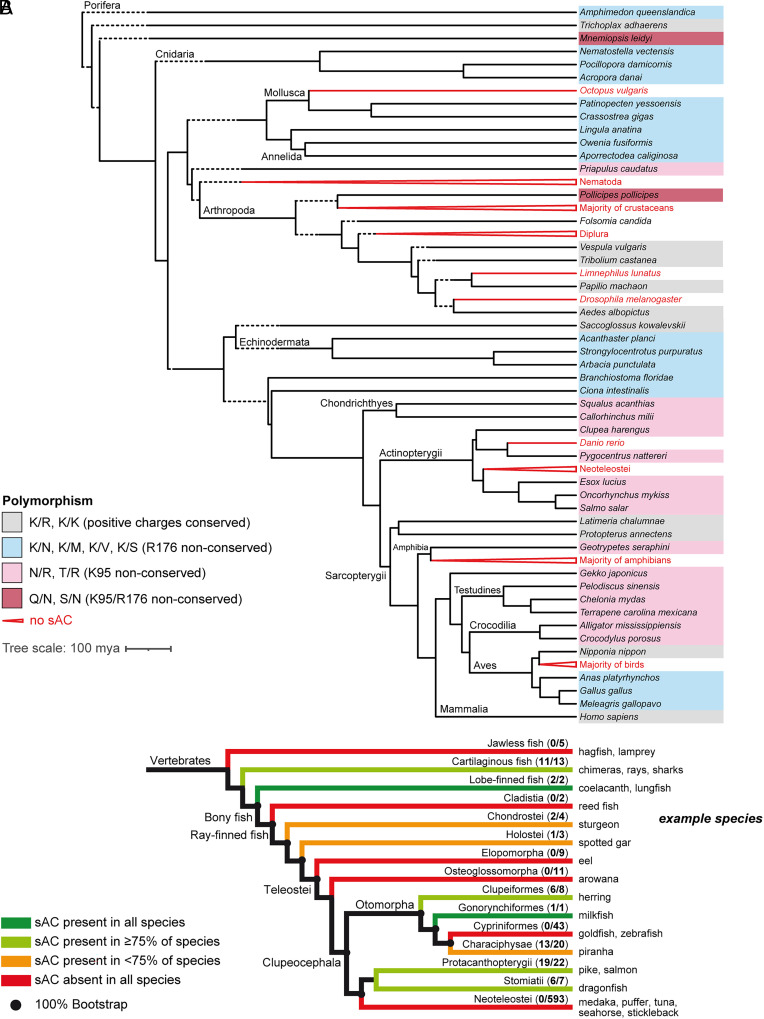
Presence of sAC and K/R polymorphism in species from 13 phyla and fish. (*A*) Phylogenetic tree created with TimeTree (timetree.org) (35) using the species given in *SI Appendix*, Table S2. Some species were omitted due to unavailability in the TimeTree database (*Rhincodon typus, Pleurobrachia bachei, Exaiptasia pallida, and Eisenia fetida*), or are aggregated to represent higher-level groups (e.g., Neoteleostei). Dashed branch segments represent an artificial extension of the tree for better legibility. Polymorphisms are marked according to color code in the figure legend, where X/Y represents the residues in the positions that are homologous to K95/R176 in *Hs*sAC, respectively. Residues are designated with 1-letter codes: K = lysine, R = arginine, N = asparagine, M = methionine, V = valine, S = serine, Q = glutamine, T = threonine. (*B*) Overview of sAC presence in fish. Cladogram of fish (taxonomy based on ref. 36), color-coded according to the fraction of sAC-possessing species. Middle column, number of species possessing sAC/number of genomes available in the respective clade at the time of investigation. For example, in cartilaginous fish, 11 of 13 searchable species possess sAC. Far right, common names of exemplary species for the respective clade.

Genomic analysis revealed that sAC genes are present in nonmammalian species from 13 phyla, excluding nematodes (Fig. 1*A* and *SI Appendix*, Table S2). Within these phyla or groups, there are notable exceptions. For instance, the sAC gene is found in many insects like wasps, beetles, butterflies, and flies but not in *Drosophila*. Similarly, many bony and cartilaginous fish possess a sAC gene, but it is missing in jawless fish (e.g., lamprey), several bony fish clades such as elopomorpha (e.g., eel), cypriniformes (e.g., zebrafish *Danio rerio*), and all clades within Neoteleostei (modern ray-finned fish like tuna, cichlids, perches, or puffer fish) (Fig. 1*B*). Finally, all but a few birds and amphibians lack sAC genes (Fig. 1*A* and *SI Appendix*, Table S2).

In human sAC (*Hs*sAC), two positively charged residues – K95 and R176 – coordinate HCO_3_^−^ (21, 37–39). These residues are conserved in all mammalian sACs, with 100 out of 100 analyzed sequences containing K95/R176 at homologous positions (*SI Appendix*, Table S3). If one or both of these residues are replaced with neutral residues, both HCO_3_^−^ sensitivity and catalytic activity are greatly diminished (21). Vacquier et al. (23) compared the sea urchin and human sAC sequences and concluded that the catalytic domain and the two residues that bind HCO_3_^−^ are conserved and that sea urchin sAC is also regulated by HCO_3_^−^ (23). However, their sequence alignment in fact shows that R176 is not conserved in sea urchin sAC; this was confirmed in our own sequence analysis and prompted us to compare the sAC sequences from species across 13 phyla (Fig. 1*A* and *SI Appendix*, Table S2).

In nonmammalian sAC, either K95, R176, or both are substituted for neutral residues, bar a few exceptions (Fig. 1*A* and *SI Appendix*, Table S2). This polymorphism falls into two main groups: one in which R176 is neutralized (R176N), while K95 is conserved; this group includes many marine invertebrates, particularly echinoderms like sea urchin; and another group where K95 is neutralized (K95N), while R176 is conserved. In bony and cartilaginous fish, the K→N substitution is common (*SI Appendix*, Table S4). Some species show substitutions of both residues, or use neutral residues besides asparagine (Fig. 1*A* and *SI Appendix*, Tables S2 and S4). Finally, the mammalian K/R genotype is found in only a few species (among fish only coelacanth and lung fish, the fish species most closely related to mammals) (Fig. 1*A* and *SI Appendix*, Tables S2 and S4). In insects, a genotype (K/K) with two positive charges is conserved (Fig. 1*A* and *SI Appendix*, Table S2).

The absence of the key residues for HCO_3_^−^ sensitivity in sAC of most nonmammals suggests regulation by factors other than HCO_3_^−^.

### The sAC of Sea Urchin and Salmon Is Regulated by pH.

We chose sAC orthologs from the sea urchin *Arbacia punctulata* (*Ap*sAC) and salmon *Salmo salar* (*Ss*sAC) to study the functional impact of R176N (sea urchin) and K95N (salmon) substitutions. Mammalian sAC, e.g., from the rat *Rattus norvegicus* (*Rn*sAC), exists as both full-length (sAC_fl_: 180 kDa) and truncated (sAC_t_: 55 kDa) isoforms (40, 41). sAC_t_ comprises two domains (C1 and C2) that contain all elements necessary for catalytic activity and regulation by HCO_3_^−^ (4). In contrast to mammalian sperm (42), sea urchin sperm contain only sAC_fl_ (29). Due to challenges to heterologously express and purify mammalian sAC_fl_, biochemical and structural studies used sAC_t_. We studied sAC_fl_ of sea urchin (*Ap*sAC_fl_), and the artificially truncated isoforms of sea urchin and salmon sAC (*Ap*sAC_t_, *Ss*sAC_t_), allowing for direct comparison with sAC_t_ from rats (*Rn*sAC_t_). The isoforms were heterologously expressed in HEK293 cells and their activity measured in cell lysates.

**Fig. 2.**
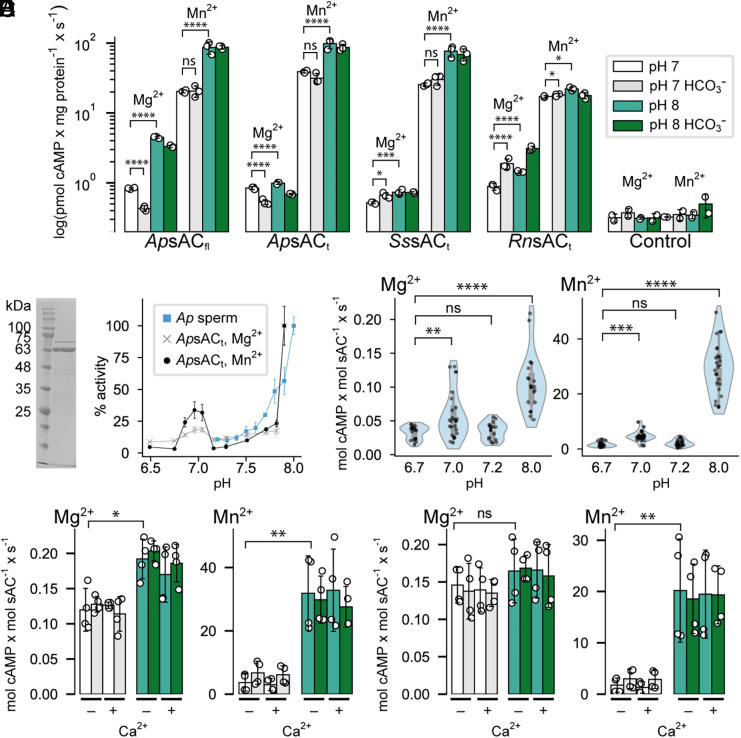
Nonmammalian sAC orthologs are pH sensitive and not activated by HCO_3_^−^ and Ca^2+^. (*A*) pH and HCO_3_^−^ sensitivity of recombinant sAC heterologously expressed in HEK cells with either MgATP (1 mM) or MnATP (1 mM) as substrate. Samples include the full-length isoform from the sea urchin *A. punctulata* (*Ap*sAC_fl_), and the truncated isoforms from *A. punctulata* (*Ap*sAC_t_), salmon (*Ss*sAC_t_), and rat (*Rn*sAC_t_). Activity was measured in HEK cell lysates at pH 7 in absence (white) and presence (gray) of 25 mM HCO_3_^−^, and at pH 8 in absence (light green) and presence (dark green) of 25 mM HCO_3_^−^. Control is nontransfected HEK cell lysate. All data mean ± SD (n = 3), statistical significance determined by two-way ANOVA followed by Tukey’s comparison test. (*B*) *Ap*sAC_t_ (1 µg) expressed in and purified from *E. coli* on 12% SDS PAGE gel, stained with Coomassie Blue (*C*) pH dependence of sAC activity in *A. punctulata* sperm (blue, dash) and of purified *Ap*sAC_t_ in the presence of 5 mM MgCl_2_ (gray) or MnCl_2_ (black, dashed line). (*D*) Turnover number of purified *Ap*sAC_t_, in the presence of 5 mM MgCl_2_ (*Upper*) or MnCl_2_ (*Lower*) from experiments using six biological replicates (samples purified from six different expressions), from each biological replicate at least two technical replicates were made. For MgCl_2_, n (total technical replicates) = 26, 20, 17, 17 for pH 6.7, 7.0, 7.2, 8.0 respectively; for MnCl_2_, n = 32, 28, 22, 22 for pH 6.7, 7.0, 7.2, 8.0. Statistical significance was determined by the Kruskal–Wallis test, followed by Dunn’s post hoc test for multiple comparisons. (*E* and *F*) Activity of purified *Ap*sAC_t_ (*E*) and *Ap*sAC_t_-N198R (*F*), at 50 nM (−) or 100 µM (+) free [Ca^2+^], respectively. Experimental conditions are color-coded as in Panel *A*. The free [Ca^2+^] was calculated with WebMaxC and adjusted with BAPTA. All data mean ± SD (n = 4) from two biological replicates, statistical significance determined by the Mann–Whitney *U* test.

Because cytosolic alkalization is important for chemotactic signaling in sea urchin sperm (43–45), we tested whether pH regulates sAC activity. Indeed, the activity of sea urchin and salmon sAC is significantly enhanced by changes in pH (Fig. 2*A*). When pH was increased from 7 to 8, cAMP production by *Ap*sAC_t_ or *Ss*sAC_t_ rose by approximately 1.2- and 1.4-fold (Mg^2+^), respectively, and 2.5- and 3.0-fold (Mn^2+^) (Fig. 2*A*). Of note, the stimulation at alkaline pH was particularly pronounced for *Ap*sAC_fl_: Stimulation was increased 5.4-fold (Mg^2+^) and 4.3-fold (Mn^2+^). The addition of HCO_3_^−^ at pH 7 slightly decreased *Ap*sAC_t_ and *Ss*sAC_t_ activity both with Mg^2+^ and Mn^2+^, and HCO_3_^−^ had no discernible effect at pH 8.0. In agreement with previous reports, HCO_3_^−^ enhanced *Rn*sAC_t_ activity twofold with Mg^2+^, and stimulation by HCO_3_^−^ was not observed when Mn^2+^ was present (3, 46). Increasing pH from 7 to 8 enhanced *Rn*sAC_t_ activity 1.6-fold with Mg^2+^ and 1.3-fold with Mn^2+^ (Fig. 2*A*). Therefore, *Rn*sAC_t_ is more sensitive to HCO_3_^−^ than to alkaline pH. In summary, the results highlight a key distinction: Recombinant *Ap*sAC_fl_, *Ap*sAC_t_, and *Ss*sAC_t_ are activated at alkaline pH but not by HCO_3_^−^, whereas *Rn*sAC_t_ is more sensitive to HCO_3_^−^ than to pH.

### Purified Sea Urchin sAC Is pH Sensitive and Not Activated by Bicarbonate and Ca^2+^.

These results contrast with prior studies, which identified a HCO_3_^−^-sensitive sAC activity in marine invertebrates and fish (11, 47–49). Our and previous experiments were performed in cell lines and lysates, which have associated complications (*SI Appendix*, Methods). Therefore, we studied the cAMP response to alkalization in purified *Ap*sAC_t._ Using purified sAC enabled direct and precise control of activation by pH, independent of HCO_3_^−^ concentration or other cellular factors. Furthermore, we also studied sAC in intact *A. punctulata* sperm; this allowed us to assess its activity under native conditions with precise adjustment of pH_i_ via the pH-clamp technique (28, 44). The *Ap*sAC_t_ was heterologously expressed in *E. coli* strain BL21(DE3) pLysS with a HA and a hexaHis tag and purified by affinity and size-exclusion chromatography (Fig. 2*B*). We obtained 113 ± 17 µg pure *Ap*sAC_t_ l^−1^ (n = 5) (Fig. 2*B*). Synthesis of cAMP was monitored by immunofluorescence (Fig. 2 *C*–*F*).

The activity of native sAC in intact sperm and of purified recombinant *Ap*sAC_t_ increased 10-fold when the pH increased from 7.2 to 8.0 (Fig. 2*C*). Examination of purified *Ap*sAC_t_ in a broader pH range revealed a biphasic pattern in the enzyme’s response. Initially, as the pH increased from 6.5 to 7.0, sAC activity rose twofold and then returned to its baseline level at pH 7.2. Between pH 7.2 to 7.8, the increase of sAC activity was relatively small. Strikingly, at pH levels above 7.8, the sAC activity surged fivefold (Fig. 2*C*). Furthermore, the activity of purified *Ap*sAC_t_ was not influenced by HCO_3_^−^ or Ca^2+^, neither alone nor in combination (Fig. 2*E*). This insensitivity persisted whether Mg^2+^ or Mn^2+^ was used as metal. In conclusion, the sAC from *A. punctulata* and *S. salar* is regulated in a manner distinct from its mammalian counterparts; it is strongly activated at alkaline conditions (high pH) and is unresponsive to HCO_3_^−^ and Ca^2+^.

Residues R176 and K95 are crucial for HCO_3_^−^ sensitivity of human sAC; their replacement with alanine nearly abolishes HCO_3_^−^-stimulated activity (21). Building on this understanding, we investigated whether *Ap*sAC_t_ acquires HCO_3_^−^ sensitivity through the N198R mutation, which is homologous to R176 in human sAC (Fig. 2*F*). However, the N198R mutant remained unresponsive to HCO_3_^−^ or Ca^2+^ under all conditions. Compared to wild-type *Ap*sAC_t_, in the presence of Mg^2+^, the N198R mutant showed similar basal activity but reduced pH sensitivity. In the presence of Mn^2+^, the mutant’s basal activity was twofold lower than that of the wild type, and its turnover number at pH 8.0 decreased from 30 to 20 mol cAMP sAC^−1^ s^−1^. These findings indicate that the N198R mutation alone does not confer HCO_3_^−^ or Ca^2+^ sensitivity, suggesting that other factors, such as the local environment of these residues, contribute to HCO_3_^−^ or Ca^2+^ sensitivity in mammalian sAC.

### The Turnover Number of *Ap*sAC_t_ Is Sufficient to Activate Downstream Targets upon Chemoattractant Binding.

Stimulation of sea urchin sperm by chemoattractants triggers an increase of pH_i_ (alkalization) by H^+^ efflux through a sperm-specific sodium/proton (Na^+^/H^+^) exchanger, known as SLC9C1 (*SI Appendix*, Fig. S1*A*). This process raises pH_i_ from a resting level of 7.2 to approximately 7.4 (ΔpH_i_ = 0.2) (44, 50, 51). Given that sAC is associated with the membrane (52), it is likely exposed to higher pH levels, potentially up to 8.0, resulting in a more significant ∆pH_i_ than in the cytosol.

To understand if this ∆pH_i_ produces enough cAMP to activate downstream targets, we determined the turnover number of purified *Ap*sAC_t_ across a pH range of 6.7 to 8.0 (Fig. 2*D*). The turnover number, which reflects the enzyme’s catalytic efficiency, peaked first at pH 7.0, decreased again to baseline levels at pH 7.2, followed by a steep increase up to pH 8. Specifically, the turnover number was 0.035 ± 0.013 mol cAMP mol sAC^−1^ s^−1^ at pH 7.2 (n = 17) and 0.106 ± 0.041 mol cAMP mol sAC^−1^ s^−1^ at pH 8 (n = 20) with MgATP as substrate (Fig. 2*D*). Considering that the flagellum of *A. punctulata* sperm contains approximately 7,000 sAC molecules (29), the pH increase from 7.2 to 8.0 could stimulate the synthesis of 497 cAMP molecules s^−1^. Within the flagellar volume of 2 fL, one molecule corresponds to a concentration of 1 nM. Therefore, 497 cAMP molecules translates to 497 nM cAMP. This concentration is well within the sensitivity range of potential targets for cAMP, such as protein kinase A (PKA) [K_D_ = 2 to 10 nM (53)] and hyperpolarization-activated cyclic nucleotide-gated channels (HCN) channels [K_1/2_ = 0.74 µM (54)]. Considering that the resting concentrations of free cGMP and cAMP in sea urchin sperm are near zero (29), this stimulated increase represents a large fold-change in cAMP levels and suggests that the chemoattractant-induced pH change is sufficient to activate downstream targets through sAC-mediated cAMP production.

### A Proposed pH-Sensitive Mechanism—Insights from AlphaFold Predictions, MD Simulations, and Mutagenesis.

We studied the structural effects of substituting K→N and R→N by modeling *Hs*sAC_t_, *Ap*sAC_t_, and *Ss*sAC_t_ using AlphaFold2 (55). The AlphaFold2 model of *Hs*sAC_t_ closely matched the crystal structure [*apo*, PDB ID 4CLF (21)], with secondary structures aligning well (rmsd = 0.56 Å) (*SI Appendix*, Fig. S2*A*). The linker region between C1-C2 domains in *Ap*sAC_t_ is twice as long as the linker in *Hs*sAC_t_. While this linker is structured in the *Hs*sAC_t_ crystal, AlphaFold2 predicts numerous conformations for the longer linker in *Ap*sAC_t_. Despite these differences, the AlphaFold2 models of *Hs*sAC_t_, *Ap*sAC_t_, and *Ss*sAC_t_ are very similar (rmsd < 1 Å) (*SI Appendix*, Fig. S2 *B* and C), indicating that the fundamental architecture of sACs is conserved.

In the crystal structures of *Hs*sAC_t_, several residues were identified that partake in the catalytic mechanism (21) (Table 1); these residues are conserved in *Ap*sAC_t_ and *Ss*sAC_t_, with the exception of the HCO_3_^−^-binding residues K95 and R176 (Table 1 and *SI Appendix*, Fig. S2*D*). In *Hs*sAC_t_, R176 is thought to function as a switch between the catalytic and the HCO_3_^−^-binding sites (21). In the unbound (*apo*) state, R176 forms an ion pair with D99. When HCO_3_^−^ binds to R176, the ion pair breaks, allowing D99 to bind a metal ion (21). By contrast, in the AlphaFold models of *Ap*sAC_t_ and *Ss*sAC_t_, the corresponding residues N198 (*Ap*sAC_t_) and R179 (*Sss*AC_t_) are oriented away from the conserved D99 residue (D121 in *Ap*sAC_t_ and D102 in *Sss*AC_t_) (*SI Appendix*, Fig. S2*D*). The R176N substitution in *Ap*sAC_t_ replaces Arg with Asn, which has a shorter side chain and different functional group. This change prevents interaction with either the conserved Asp in the catalytic domain or within the HCO_3_^−^-binding site. Similarly, the K95N substitution in *Ss*sAC_t_ hinders HCO_3_^−^-binding due to the shorter side chain of Asn. This analysis supports the conclusion that K95 and R176 are crucial for HCO_3_^−^-binding, and replacing these residues results in a loss of sensitivity to HCO_3_^−^.

**Table 1.** Functional amino-acid residues in *Hs*sAC and homologous residues in *Ap*sAC and *Ss*sAC

Description^*^	*Hs*sAC	*Ap*sAC	*Ss*sAC
Linker	216–249	239–330	199–251
Catalytic domain 1 (C1)	4–215	26–238	8–198
Catalytic domain 2 (C2)	250–462	331–542	252–467
Binds bicarbonate in *Hs*sAC	Arg176	Asn198	Arg179
Binds bicarbonate in *Hs*sAC	Lys95	Lys117	Asn98
Recognizes ATP base (via H_2_O)	Lys334	Lys414	Lys335
Coordinates metal ion, interacts with Lys176	Asp99	Asp121	Asp102
Coordinates metal ion	Asp47	Asp69	Asp50
PP_i_ “receptor“	Lys144	Lys166	Lys147
Interacts with α-phosphate	Arg416	Arg496	Arg418
Product release (steric clash)	Asn412	Asn492	Arg414
C1-ß2/3 loop	97–99 (AGD)	119–121 (AGD)	100–102 (AGD)
C2-ß2/3 loop	338–341 (FDKG)	418–421 (FDKG)	339–342 (FDKG)

^*^Functional descriptions refer to *Hs*sAC only. In *Ap*sAC and *Ss*sAC, the function of the homologous residues has not yet been studied by high-resolution structures.

Although these observations help to understand how HCO_3_^−^ regulation was lost, the mechanism for gaining pH regulation remains unclear. One hypothesis is that changes in pH alter the protonation state of amino acid side chains in sAC. This could induce a local conformational change, acting as a “switch” similar to R176 in *Hs*sAC_t_ (21). It is important to note that the acid dissociation constant (pK_a_) of an amino acid side chain is influenced by its electrostatic environment within a protein, potentially differing from its pK_a_ in aqueous solution.

**Fig. 3.**
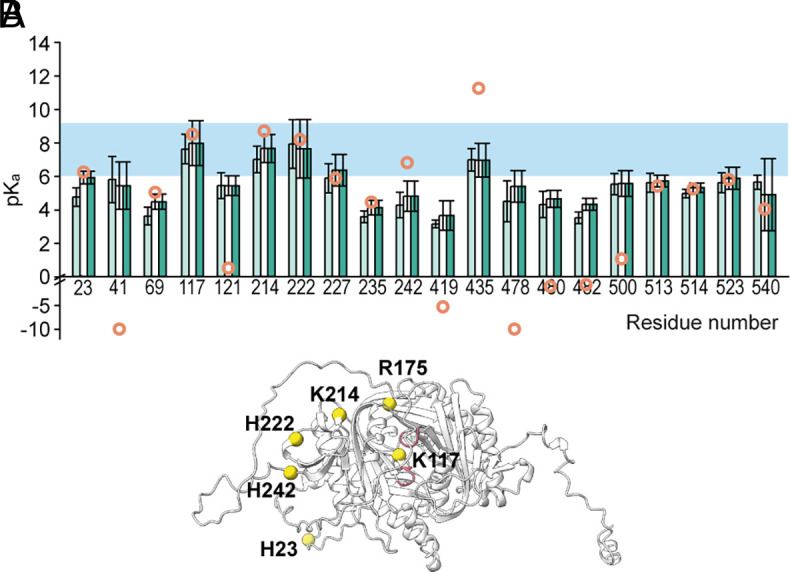
pK_a_ predictions of *Ap*sAC_t_. (*A*) 20 pK_a_ values predicted by constant pH MD simulations with AMBER at pH 6 (light green), 7 (white), and 8 (dark green), overlaid with pK_a_ values from Karlsberg2^+^ (open orange circles, *SI Appendix*, Fig. S3). All histidine residues were titrated, in addition K117 and K214, which were identified by Karlsberg2^+^ program. All data mean ± SD (n = 20). (*B*) AlphaFold2 model of *Ap*sAC_t_. Residues with pK_a_ values between 6 to 9, as predicted by Karlsberg2^+^, are indicated by yellow spheres, and catalytic loops are colored pink.

To investigate this, a computational titration of *Ap*sAC_t_ was performed using Karlsberg2^+^ (56, 57). We focused on pK_a_ values from 6 to 9, a range considered physiologically relevant. The analysis predicted seven residues (M1, H23, K117, R175, K214, H222, and H242) with titratable side groups within this pH range (Fig. 3*A* and *SI Appendix*, Fig. S3*A*). One of these residues is the N-terminal residue, five are located on the C1 domain, and one is near the catalytic site (Fig. 3*B*). Notably, residue K117 in *Ap*sAC_t_, which is homologous to K95 in *Hs*sAC_t_, was predicted to have a pK_a_ of 8.54. This value suggests that K117 responds to proton concentrations two orders of magnitude higher than what is typically observed for a lysine side chain in solution (pK_a_ = 10.5). Therefore, the pK_a_ of K117 could be responsible for conferring pH sensitivity to *Ap*sAC_t_. By contrast, the pK_a_ of K95 in *Hs*sAC_t_ is 10.14 (*SI Appendix*, Fig. S3*B*), indicating that this residue is fully protonated under physiological pH conditions, supporting its proposed role as a “positively charged HCO_3_^−^ docking site” (21).

In contrast to sea urchin sAC, K95 is not conserved in *Ss*sAC_t_. Additionally, R179 in *Ss*sAC_t_—the residue homologous to R176 in *Hs*sAC_t_—is not predicted to have a pK_a_ in the physiological range (*SI Appendix*, Fig. S3*C*), and, therefore, is unlikely to confer pH sensitivity. Instead, in *Ss*sAC_t_ six residues (M1, D50, E73, D91, H242, and H539) are predicted to have pK_a_ values within this range; of these residues, only D50 is conserved in *Ap*sAC_t_. Thus, the pH sensitivity of *Ss*sAC_t_ likely results from residues other than those in *Ap*sAC_t_.

We further examined the pK_a_ of residues in *Ap*sAC_t_ using Constant pH Molecular Dynamics (CpHMD) simulations (58, 59). The results were analyzed by the AMBER Tools package cphstats and TTclust (60, 61). This technique is valuable because changes in the protonation state of one residue can influence the protonation state of nearby residues, subsequently affecting their orientation and the broader H-bond network. CpHMD simulations allow the protein model to adapt to these changes in protonation state. For this analysis, we conducted twenty 100 ns simulations to minimize the risk of inaccurate pK_a_ approximations from results trapped in a local energy minimum. Due to the limitations in the number of residues that can be titrated simultaneously, we selected all histidine residues for titration, along with those residues identified by the Karlsberg2^+^ screening. Analysis of the simulations revealed that the C1 and C2 domains exhibited only minor positional fluctuations (root mean-squared fluctuation < 4 Å), whereas the linker region displayed large fluctuations (*SI Appendix*, Fig. S4). The calculated pK_a_ values showed minimal variation at pH 6, 7, and 8 (Fig. 3*C*). Notably, the pK_a_ values of residues K117, H222, K214, and H435 fell within the pH 6 to 9 range. Significantly, three of these residues (K117, H222, and K214) were also highlighted in the initial Karlsberg2^+^ screening. Consequently, we tested the contribution of these specific residues to AC activity through mutagenesis experiments.

To study the roles of K117, H222, and K214 in the activity of *Ap*sAC_t_, each was mutated to alanine (removing effects of a longer side chain), arginine (mimicking a protonated, positively charged side chain), and either asparagine or glutamine (mimicking a deprotonated, neutral side chain). The activity of the mutants was assessed at four pH values ranging from 6.7 to 8.0. This was done to determine if pH regulation was maintained and to compare their basal activity level at pH 6.7 with that of the wild-type *Ap*sAC_t_. Measuring basal activity was an important control to ascertain if the mutations generally affected or abolished enzyme activity.

**Fig. 4.**
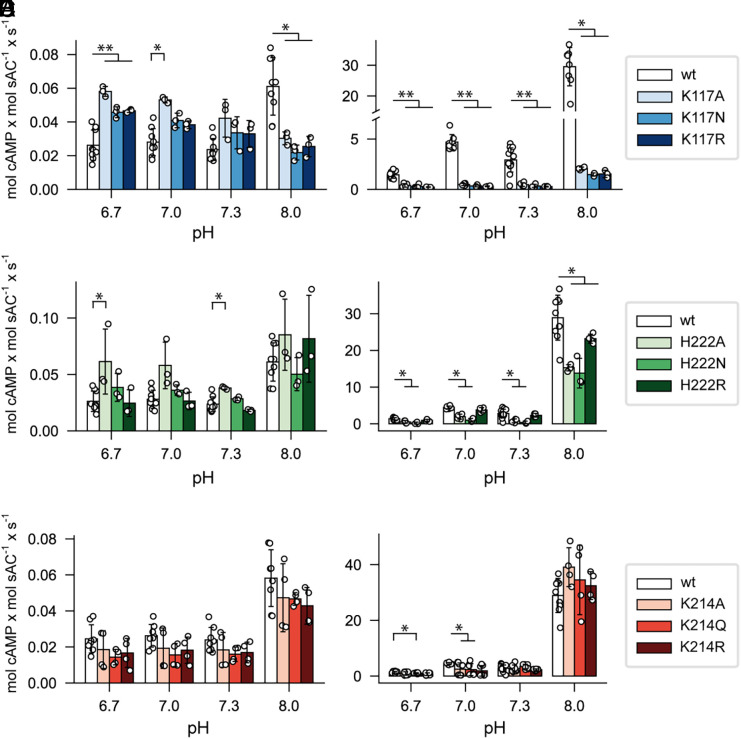
Activity of *Ap*sAC_t_ mutants. Activity was measured in the presence of 5 mM MgCl_2_ or MnCl_2_. (*A* and *B*) Activity of *Ap*sAC_t_-K117 mutants in MgCl_2_ (*A*) and MnCl_2_ (*B*). (*C* and *D*) Activity of *Ap*sAC_t_-H222 mutants in MgCl_2_ (*C*) and MnCl_2_ (*D*). (*E* and *F*) Activity of *Ap*sAC_t_-K214 mutants in MgCl_2_ (*E*) and MnCl_2_ (*F*). Open circles represent individual measurements of technical replicates from three biological replicates. At each pH value, n (total technical replicates) = 8 for wild-type *Ap*sAC_t_ and n = 3 to 8 for each mutant, error bars represent SD. Statistical significance of each mutant vs. wild type at each pH was assessed using the Mann–Whitney *U* test, followed by Benjamini–Hochberg correction to control the false discovery rate.

In the presence of Mg^2+^, replacing K117 (homologous to K95 in *Hs*sAC) significantly impaired activity at pH 8.0 (Fig. 4*A*), yet the mutants exhibited a twofold higher basal activity (pH 6.7 to 7.0) compared to wild-type *Ap*sAC_t_ (Fig. 4*A*), indicating that the enzyme remained active. In the presence of Mn^2+^, the activity of these K117 mutants was reduced 20-fold at all pH levels compared to the wild type (Fig. 4*B*). However, the stimulation of activity at pH 8.0 was maintained (activity increased 10-fold as the pH increased from 6.7 to 8.0). This observation suggests that the metal ion functions as a Lewis acid in the pH-sensitive mechanism. Given that Mn^2+^ is a stronger Lewis acid than Mg^2+^, we propose that Mn^2+^ more effectively polarizes bonds not only in the ATP molecule (21, 62) but also in surrounding water molecules, thereby facilitating proton exchange. Consequently, the pH-sensitive mechanism still operates with Mn^2+^ in the K117 mutants, albeit at lower activity levels. These results highlight a critical role for K117 in the pH regulation of *Ap*sAC_t_.

In the presence of Mg^2+^, the H222 mutants exhibited activity similar to the wild-type *Ap*sAC_t_, indicating H222 is not crucial for the pH-sensitive mechanism under these conditions (Fig. 4*C*). However, when Mn^2+^ was present, the activity of the H222A and H222N mutants decreased twofold across all pH values, whereas the activity of the H222R mutant remained similar to the wild type (Fig. 4*D*). This difference in activity with Mn^2+^ vs. Mg^2+^ can be attributed to Mn^2+^ being a stronger Lewis acid, thereby exerting a greater effect on the protonation states of residues within the protein. Indeed, H222, located at the end of the C1 domain and beginning of the linker, is distant from the active site (Fig. 3*B* and Table 1). These findings suggest that the specific protonation state of multiple residues are important for optimal sAC activity.

The K214 mutants showed activity levels largely consistent with the wild-type *Ap*sAC_t_ in the presence of both Mg^2+^ or Mn^2+^ (Fig. 4 *E* and *F*). Therefore, K214 is not essential for the pH regulation of *Ap*sAC_t_.

The structural consequences of the N198R and K117N mutations on *Ap*sAC_fl_ were explored in silico using effective strain analysis (63) implemented in MATLAB (*SI Appendix*, Fig. S5). This analysis employed the full-length *Ap*sAC to examine the potential widespread effects of these mutations throughout the protein. The results indicate that the N198R mutation led to increased effective strain throughout the entire *Ap*sAC_fl_ structure, consistent with the experimentally observed reduction of activity. Conversely, the K117N mutation induced almost negligible effective strain when compared to wild-type *Ap*sAC_fl_. This suggests that the lower activity observed with the K117N mutation is due to subtle changes within a H-bond network rather than large structural alterations.

To summarize, K117 is important for the pH-sensitive mechanism in *Ap*sAC (Fig. 4 *A* and *B*). However, even when K117 is substituted, the pH dependence remains in the presence of Mn^2+^ (Fig. 4*B*), and mutations of N198 and H222 also affect the pH sensitivity of *Ap*sAC_t_. Altogether, this suggests that a H-bond network supports the pH-regulated mechanism. In silico predictions of *Ap*sAC stability with these mutations revealed that the K117N mutation does not alter *Ap*sAC, whereas the N198R mutation induces internal strain within *Ap*sAC (*SI Appendix*, Fig. S4). These results offer an explanation for the lower activity of the N198R mutant and support the concept that the pH sensitivity originates from a H-bond network.

### Simulation of Spawning of *A. punctulata* Sperm Produces Hyperpolarization, Alkalization, and a Rise of cAMP.

Having established that recombinant sAC of *A. punctulata* and *S. salar* is activated at alkaline pH, we investigated the connections between the pH regulation of sAC and the physiological events that trigger alkalization in sperm during spawning. Generally, sperm are inactive in seminal fluid (SF) and become motile after release, a process linked to alkalization (for review see refs. 24, 25, and 64–66). Two main mechanisms explain the activation of motility in sea urchin sperm: either the direct activation of motor proteins by alkaline pH_i_ (67) or the phosphorylation of axonemal proteins, possibly dyneins, by cAMP-stimulated PKA (26, 68). Given that pH_i_ and cAMP are directly tied through the pH-regulated sAC, and considering the two-step pH dependence of *Ap*sAC, we reasoned that the transition from SF to sea water alkalizes sperm, thereby raising cAMP levels and initiating motility. To test this, we examined several factors i) the pH and ionic composition of *A. punctulata* SF; ii) the pH_i_ of sperm in SF and its change after spawning into artificial sea water (ASW) or artificial seminal fluid (ASF, which is ASW containing 27 mM K^+^ at pH 6.7); iii) changes in membrane potential (V_m_), pH_i_, and cAMP levels after spawning into ASW; and finally iv) changes in motility after spawning into various solutions, including ASF and ASW at different pH levels and K^+^ concentrations ([K^+^]).

The ion concentrations in SF from *A. punctulata* (*SI Appendix*, Table S5) or other sea urchin species (69) are nearly identical to sea water, with the notable exceptions of K^+^ and pH. Specifically, the [K^+^] in SF was 25.7 ± 2.1 mM (n = 5) compared to 9 mM in ASW. The pH of both undiluted, freshly spawned semen (sperm cells plus seminal fluid) and of seminal fluid was 6.8 ± 0.2 (n = 5), significantly lower than the ASW pH of 7.8.

Using the null-point pH-clamp technique (44), we determined the resting pH_i_ of sperm in ASF to be 6.84 ± 0.02 (n = 3) (*SI Appendix*, Fig. S6 *A* and B), similar to the pH of seminal fluid. When sperm cells were diluted into ASW, their pH_i_ rose to 7.2 (*SI Appendix*, Fig. S6*C*) (44). These findings indicate that sperm undergo alkalization upon release into seawater. Importantly, this ∆pH_i_ range (6.84 to 7.2) coincides with the first peak of cAMP synthesis when the pH dependence of purified sAC was studied (Fig. 2*C*).

**Fig. 5.**
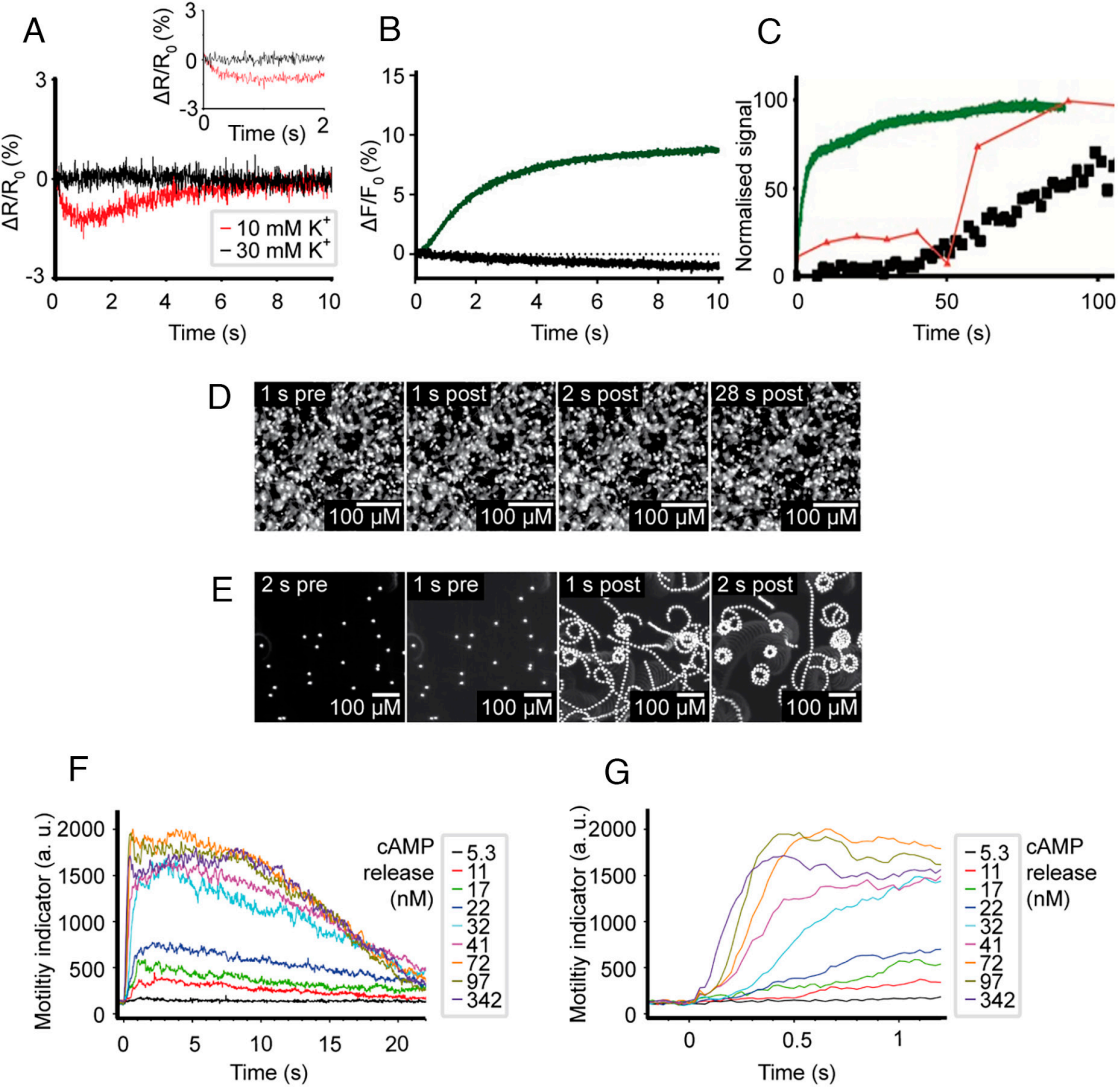
Cellular signaling events during spawning and activation of sperm. Panels *A*–*D*, sea urchin sperm; panels *E*–*G*, salmon sperm. (*A*) Changes in V_m_ measured with the potentiometric probe Di-8-ANNEPS after 1:2 mixing of *A. punctulata* sperm in ASF with 0KASW, yielding final concentration of 10 mM K^+^ (red), and 30 KASW (black). Inset: enlarged time scale. (*B*) Changes in pH_i_ measured with pHrodo Red after 1:2 mixing of *A. punctulata* sperm in ASF with 0KASW (green) or 30KASW (black). The pH_i_ signal has been inverted. (*C*) Comparison of the time course of pH_i_ response (green), cAMP increase (red), and motility (black) after dilution by 0KASW of *A. punctulata* sperm incubated in ASF. (*D*) Trajectories of sperm from *A. punctulata* in ASF from Movie S1, recorded 1 s before (*Left*) and 1, 2, and 28 s after (*Right*) release of cAMP by light. Experiments were performed at least three times with similar results. (*E*) Similar experiment as (*D*) for salmon sperm, recorded 1 and 2 s before (*Left*) and after (*Right*) release of cAMP by light. (*F*) Initiation of motility by UV light flashes of different energy in salmon sperm bathed in salmon ASF and loaded with DEACM-caged cAMP (20 μM), which raised cAMP concentrations from 5.3 nM to 342 nM. Each data point represents the mean from three animals and 6 to 9 experiments. (*G*) Extended timescale that illustrates the dose-dependent latency of the motor response. UV flash was delivered at t = 0.

To reveal the cellular events and underlying mechanisms during spawning of *A. punctulata* sperm, we emulated this process using rapid-mixing techniques (28), while monitoring changes in V_m_ and pH_i_ with fluorescent indicators. Initially, sperm in ASF were loaded with the respective fluorescent probes and then diluted with ASF. This diluted sperm suspension was rapidly mixed 1:2 in a stopped-flow device with either K^+^-free ASW (0KASW, pH 8.0), causing the extracellular [K^+^] ([K^+^]_o_) to drop to 10 mM, or with ASW containing 30 mM K^+^ (30KASW, pH 8.0), which kept [K^+^]_o_ unchanged. For both experimental conditions, the pH_o_ was increased from 6.7 to 7.8. Given that [K^+^]_o_ controls the resting V_m_ of sea urchin sperm (44, 70), it was predicted that sperm would hyperpolarize during spawning due to the lower [K^+^] in ASW. Indeed, mixing with 0KASW evoked a rapid, transient hyperpolarization (Fig. 5*A*), accompanied by a pronounced intracellular alkalization (Fig. 5*B*). Both the hyperpolarization and alkalization were entirely prevented when sperm were mixed with 30KASW (Fig. 5 *A* and *B*). This observation is remarkable, because it indicates that protons do not redistribute passively across the sperm membrane via conventional Na^+^/H^+^ exchange; rather, a change in V_m_ is necessary to activate the voltage-gated Na^+^/H^+^ exchanger SLC9C1 (45).

We then examined whether the observed changes in V_m_ and pH_i_ during spawning influence cAMP levels after mixing with ASW. The baseline cAMP level of sperm in ASF was extremely low (0.23 ± 0.1 pmol × 10^8^ cells^−1^, n = 5, data from *SI Appendix*, Fig. S6*D* at t = 0). Of note, prior studies reported higher baseline cAMP levels [25 pmol × 10^8^ cells^−1^; (71)], because cAMP had been determined in diluted sperm/ASW suspensions preincubated with IBMX, a phosphodiesterase inhibitor. Upon dilution into ASW, cAMP levels rose approximately fivefold (15.1 ± 4.0 pmol × 10^8^ cells^−1^) with a half-time of approximately 50 s (Fig. 5*C*). We present three examples of individual time courses and two controls in *SI Appendix*, Fig. S6*D*.

In summary, spawning first triggers a change in V_m_, followed by a rise of pH_i_ that finally stimulates cAMP synthesis.

### Sperm Motility Is Initiated by Alkalization and a Rise of cAMP.

Next, we investigated how alterations in [K^+^]_o_ and pH_i_ during spawning influence sperm motility in the sea urchin *A. punctulata*. When “dry” sperm were diluted into ASW (1:200), most cells became motile (87.5 ± 5.3%, n = 3, 740 cells). By contrast, dilution into ASF did not activate motility, demonstrating that the pH and ionic composition of ASF keeps sperm quiescent, rather than an unidentified inhibitory factor in natural SF. Activation of motility in ASW was not immediate, commencing after approximately 40 s (Fig. 5*C*). This delay likely reflects the time needed for cAMP synthesis, activation of PKA, and the subsequent phosphorylation of motor proteins.

Further experiments explored whether changes in either [K^+^]_o_ or pH alone, upon dilution from ASF to ASW, were sufficient to activate motility. Diluting sperm into ASF at pH 7.8, or into ASW at pH 6.7, or into ASW containing high [K^+^]_o_ (30KASW), resulted in only a small fraction (4 to 15%) of sperm becoming motile. Specifically, motility rates were 9.9 ± 4.1% in ASW 6.7 and 17.6 ± 14.8% in 30KASW (n = 3; 490 to 536 cells). This demonstrates that the difference in pH_o_ between SF and ASW alone is insufficient to initiate sperm motility. A concurrent decrease of [K^+^]_o_ is essential. Therefore, the same mechanisms underlie the stimulation of cAMP synthesis by sAC and the initiation of sperm motility.

In summary, the proposed sequence of events upon spawning of sea urchin sperm is as follows: First, [K^+^]_o_ decreases, then sperm cells hyperpolarize, Na^+^/H^+^ exchange by SLC9C1 is activated, the subsequent intracellular alkalization activates the pH-regulated sAC. This entire sequence of events activates motility. This model reconciles previous hypotheses suggesting that either a change in cAMP or pH_i_ independently triggers motility, by demonstrating their interconnected roles in the activation process.

To test if cAMP directly initiates motility in *A. punctulata* sperm, we used caged cAMP. Immotile *A. punctulata* sperm were placed in sea urchin ASF and loaded with DEACM-caged cAMP. Upon exposure to UV light, *A. punctulata* sperm do not become motile upon the release of cAMP (Fig. 5*D* and Movie S1). The same result was obtained using a different caged compound, BECMCM-caged cAMP. This finding suggests that cAMP alone may be not sufficient to initiate motility in sea urchin sperm; PDEs are ferocious enzymes, and cAMP produced by the short light pulse may be hydrolyzed, like cGMP, with τ_hydrolysis_ ≤ 350 ms (29)–too short for PKA activation and the subsequent phosphorylation of motor proteins. We assume that concomitant changes in V_m_ or pH_i_, are necessary. Indeed, it has been shown that, in sea urchin, dynein ATPases are activated at alkaline pH (67, 72, 73). Thus, the activation of dynein motor ATPases may also differ between salmon and sea urchin sperm.

To test if cAMP initiates motility in fish, we repeated these experiments using caged cAMP in salmon sperm. Salmon sperm typically become motile when released into freshwater (25). Upon exposure to UV light, sperm became motile depending on the amount of cAMP released by the light flash (Fig. 5 *E* and *F* and Movie S2). At high cAMP concentrations, 412 out of 429 sperm (96%) responded and initiated movement within as little as 100 ms (Fig. 5*G*). Following this cAMP increase, sperm swam on curved paths (Fig. 5*E*) for approximately 20 s before becoming immotile again (Fig. 5*F*). This transient motility is reminiscent of the motility observed in sperm from various other fish species after spawning (25).

These findings offer three key insights: i) Many marine organisms may share a common mechanism for initiating motility, involving a rise of cAMP, although distinct differences exist as illustrated by sea urchin and salmon sperm. ii) The experiment using caged cAMP directly shows that a cAMP increase is both necessary and sufficient to initiate motility in salmon, whereas concomitant changes in V_m_ or pH_i_ may be necessary to initiate swimming in sea urchin sperm. iii) Motility in salmon is transient because, after the initial rise, cAMP might be broken down by phosphodiesterases (PDE), and the phosphorylation events that enable motility may be reversed by phosphatase activity, leading to cessation of motility.

## Discussion

We show that sAC orthologs in metazoans fall into functionally distinct subfamilies. Mammalian sAC is directly activated by HCO_3_^−^ and Ca^2+^. By contrast, sAC in sea urchin and salmon is activated by alkaline pH but not HCO_3_^−^ and Ca^2+^. The lack of HCO_3_^−^ responsiveness can be attributed to amino acid substitutions in the HCO_3_^−^-binding pocket. Specifically, in pH-sensitive sAC isoforms, at least one of two key residues that coordinate HCO_3_^−^ in mammalian sAC is not conserved. The K→N and R→N polymorphisms observed in nonmammalian species across 13 phyla suggest that their sAC orthologs similarly lack HCO_3_^−^ and Ca^2+^ regulation and are instead directly controlled by pH. It follows that the nonmammalian sAC senses changes in pH directly rather than indirectly through adjustments of the CO_2_/HCO_3_^−^/H^+^ equilibrium.

These results help clarify how sAC is activated in low-HCO_3_^−^ environments, a topic that has been controversial. While direct pH activation was previously observed in sAC from the sea urchin *S. purpuratus* (74), and a link between pH_i_ and cAMP synthesis was noted in sperm of early-branching metazoans like corals and even in plant sperm (9, 47, 75, 76), this relationship was either tacitly or explicitly attributed to the CO_2_/HCO_3_^−^/H^+^ equilibrium due to reports of HCO_3_^−^ stimulating cAMP synthesis in corals, sea urchins, trout, and dogfish (11, 47–49) (see *SI Appendix*, Table S1 and discussion therein).

We demonstrate here that in sea urchin and salmon, sAC is directly regulated by pH, and neither HCO_3_^−^ nor Ca^2+^ affect its activity. Our result for salmon contrasts with previous findings in trout, where sAC was reported to be HCO_3_^−^-sensitive but not pH sensitive (49), despite the sAC sequences of trout and salmon showing 99% identity (trout *Salmo trutta*: XP_029597498.1; *Salmo salar* XP_0140072021). Critically, the HCO_3_^−^-binding residues of mammalian sAC are also not conserved in trout. By contrast, properties of mammalian sAC are remarkably similar across the mouse, rat, and humans, despite their sequences being more divergent than those between trout and salmon (see e.g., ref. 46).

Reconciling previous reports with our results requires the consideration of several factors. First, the relationship between HCO_3_^−^ and pH_i_ is inherently linked through the CO_2_/HCO_3_^−^/H^+^ equilibrium, meaning HCO_3_^−^ and pH_i_ cannot be independently altered. Second, in experiments working with cells, the mechanism by which HCO_3_^−^ enters the cytosol determines the experimental outcome. If HCO_3_^−^ enters via HCO_3_^−^ transporters or anion channels, the subsequent equilibrium adjustment would cause cellular alkalization, thereby activating the pH-sensitive sAC. Conversely, if HCO_3_^−^ is synthesized intracellularly from CO_2_ and H_2_O by carbonic anhydrases, the cytosol would acidify, thereby decreasing sAC activity (for a comprehensive discussion, see ref. 18). However, in marine environments, HCO_3_^−^ levels are low, and HCO_3_^−^ does not alkalize the cell in external fertilizers; we show that in *A. punctulata* sperm, a decrease in [K^+^]_o_ upon spawning causes this alkalization. Third, HCO_3_^−^ can act as a chaotropic agent, potentially altering protein structures and water layers at the membrane/water interface in subtle ways, which could disrupt H-bond networks and consequently affect sAC activity. Finally, unnoticed pH shifts in HCO_3_^−^-containing solutions may account for contradictory findings. HCO_3_^−^ solutions can alkalize, even when stored for short times in contact with atmospheric pCO_2_, as CO_2_ degasses into the air (77). Indeed, this phenomenon was observed in our own experiments: When testing the effect of HCO_3_^−^ at pH 7.0, the pH of the assay mixture increased to 7.2 within 10 to 20 min required to prepare other experimental components. Given the pronounced pH sensitivity of *Ap*sAC, even minor pH deviations can significantly alter sAC activity. Future studies should reexamine HCO_3_^−^ sensitivity in species with K→N or R→N substitutions.

The two-step pH dependence reveals a dual functionality of sAC in sea urchin sperm. First, cAMP levels rise transiently upon alkalization from spawning, which triggers motility (Fig. 5*C*). A second rise in cAMP occurs with further alkalization following chemoattractant stimulation (71). Thus, sAC regulates two distinct functions across different pH ranges, each activated by separate biological events: spawning and chemotaxis. Although both spawning and chemotaxis involve hyperpolarization followed by alkalization, their triggering mechanisms differ. During spawning, hyperpolarization results from a decrease in [K^+^]_o_, whereas during chemotaxis, it is caused by opening of K^+^ channels and subsequent K^+^ efflux (70, 78).

The pH sensitivity and membrane localization of *Ap*sAC (5, 46, 52), along with its potential link to the SLC9C1, hint at a broader regulatory mechanism that may be important in cellular signaling. The sharp increase in sAC activity between pH 7.8 and 8.0 suggests its pH sensitivity is finely tuned to the local environment at the membrane–cytosol interface; here, the local pH differs from that of the cytosolic bulk and is particularly influenced by the Na^+^/H^+^ exchange activity of SLC9C1. A previous study reported that the SLC9C1 physically associates to sAC in the sea urchin *Strongylocentrotus purpuratus* (79). Thus, in sea urchin sperm, the pH-sensitive sAC and a cAMP-sensitive SLC9C1 regulate each other: A voltage change triggers alkalization through the action of SLC9C1. This alkalization stimulates sAC to synthesize cAMP. In turn, cAMP binds to a specific site on the SLC9C1, shifting its voltage-dependent activation (45) (*SI Appendix*, Fig. S1*B*) and causing major conformational changes (80, 81). This model also explains previous findings that voltage indirectly controls cAMP synthesis in sea urchin sperm (82).

In mammals, a similar mechanism of reciprocal control between sAC and SLC9C1 may exist. Male mice lacking SLC9C1 produce nonmotile sperm and are infertile (42, 83). The motility and infertility can be reversed by increasing levels of cAMP or its analogues (42, 84, 85), indicating a physical or functional link between SLC9C1 and sAC (42). Specifically, sperm from SLC9C1-knock-out mice lack the full-length sAC_fl_ (42), suggesting that SLC9C1 is necessary for the expression of sAC and that sAC_fl_ is the physiologically relevant form. Although the mechanisms of regulation of the individual proteins differ across species (86), the conserved expression of sAC, SLC9C1, and CatSper in many species hints at an important interaction between these three proteins (9, 87).

The mechanism by which sAC senses pH may involve a hydrogen-bond network, similar to how protons are translocated across membranes in H^+^-transporting pumps or channels like bacteriorhodopsin or the Hv1 proton channel. In such networks, H^+^ movement occurs through a series of side groups and water molecules, rather than relying on a single residue as the sole H^+^ donor or acceptor. This makes it challenging to identify the network components through single-residue mutagenesis. Two examples are the proton channel H_v_1 (88) and the archaeal H^+^ pump bacteriorhodopsin (89–91). This H-bond network could explain why a specific residue (K117) is important for the pH-sensing network in *Ap*sAC, while its equivalent residue Asn98 in *Ss*sAC is not. At any rate, the biphasic response of *Ap*sAC to pH also suggests that multiple sites or H-bond networks control activity across two different pH ranges.

The pH sensitivity of nonmammalian sAC offers a distinct amplification mechanism compared to tmACs. The amplification using sAC may benefit from two factors: A pH change is sensed by *all* 7,000 sAC molecules (29) tethered to the sperm membrane, because changes in pH produced by H^+^/Na^+^ exchange via SLC9C1 spread laterally along the membrane surface. Furthermore, during SLC9C1 activity, the local ∆pH at the membrane/cytosol interface exceeds the bulk ∆pH, potentially leading to a larger cAMP increase. Additionally, the cAMP-binding sites of SLC9C1 and HCN channels are situated near the membrane surface, placing the cAMP source and cAMP targets close together. These findings underscore that chemotactic signaling in sperm primarily happens at the membrane surface (29).

Climate change contributes to the progressive acidification of the oceans. While this issue was not examined here, extensive experimental evidence from various reports indicates that ocean acidification impairs reproduction in the sea, with notable impacts observed in echinoderms and fish (92–95). The pH regulation of sAC in sperm is suggested as one of several potential mechanisms by which declining pH levels could negatively affect reproduction success in marine ecosystems.

## Methods

### Bioinformatics.

Bioinformatic searches were performed using BLAST (blastp and tblastn) against publicly available genomes (NCBI, November 2021). Candidate sAC proteins were identified based on homology and validated through phylogenetic analysis (MAFFT alignment, PhyML tree building). Further details are provided in the *SI Appendix*.

### Computational Methods.

The pK_a_ values of residues in the homology model of *Ap*sAC_t_, provided by C. Steegborn, were predicted with Karlsberg2^+^ (57). Modeling in AlphaFold2 v2.2.4 was performed within the ColabFold v1.5.2 pipeline (96), with multiple sequence alignments generated using MMSeqs2 (97). Constant pH MD simulations were performed on the AlphaFold model of *Ap*sAC_t_ with AMBER 22 with the Amber ff14SB force field (59, 60). All histidine residues and D69, K117, D121, K214, and D419 were titrated (20 total). Twenty replicates were made at pH 6, 7, and 8 each. The trajectories were analyzed with ttclust (61), cphstats, and AmberTools22 (60). Karlsberg2^+^, AlphaFold2, and the effective strain analysis are described in *SI Appendix*.

### Cloning of Full-Length and Truncated sAC.

The full-length sequence of *Ap*sAC was obtained by PCR amplification on an *A. punctulata* testis library. The truncated sACs from *A. punctulata*, *R. norvegicus*, and *Salmo salar* were constructed by successive PCRs. Further details are provided in the *SI Appendix*.

### Generation of HEK293 Cell Lines Stably Expressing *Ap*sAC_t_, *Ss*sAC_t_, or *Rn*sAC_t_.

HEK293 cells were electroporated with the respective plasmids using the Neon 100 Kit (Invitrogen, Carlsbad, USA) and a MicroPorator (Digital Bio). HEK293 cells stably overexpressing *Rn*sAC_t_ were as described (98). Further details are provided in the *SI Appendix*.

### Expression and Purification of *Ap*sAC_t_.

The PCR fragment of *A. punctulata* sAC_t_ (aa 1 to 577) with a C-terminal HA and His tag was cloned into a pET21a vector. This construct was used to generate mutants via the QuikChange Protocol (99) (*SI Appendix*, Table S6). The construct was expressed in BL21(DE3)pLysS cells. *Ap*sAC_t_ was purified from cell lysate by cobalt affinity chromatography and size-exclusion chromatography. Further details are provided in the *SI Appendix*.

### Measurement of sAC Activity and Quantification of cAMP Content in Sperm and Cell Lines.

The cAMP synthesis of intact sperm from sea urchin on the subsecond time scale was followed using the stopped-flow instrument at room temperature. Experiments on the seconds to minutes time scale were performed on the bench.

cAMP production by heterologously expressed sAC in HEK293 lysates or by purified *Ap*sAC_t_ was induced by applying Na_2_ATP (Sigma Aldrich) and MgCl_2_ or MnCl_2_ in isotonic HEPES buffer. The final pH in the assay tube was confirmed using a Greisinger GMH 3530 pH microelectrode. Reaction time was 10 min. cAMP levels were determined using the CatchPoint cAMP Fluorescent Assay Kit (Molecular Devices, San Jose, USA). The cAMP content of intact HEK293 cells stably expressing *Rn*sAC_t_ was determined by an accumulation assay (98), using Correlate-EIA Direct Assay. Further details are provided in the *SI Appendix*.

### Simulation of Spawning in the Stopped-Flow Device.

To simulate spawning, dye-loaded sperm suspensions in ASF or ASW were rapidly diluted in the stopped-flow device into various solutions and changes in pH_i_ and V_m_ were measured using the respective fluorescent dyes (BCECF-AM or pHrodo Red-AM for pH, and FluoVolt or Di-8-ANNEPS for V_m_) (28). Dye-loading of the sperm and the detection of changes in emitted fluorescence is detailed in the *SI Appendix*. The techniques are also comprehensively described in ref. 28.

### The pH-Clamp Method.

The pH_i_ sensitivity of sAC in intact *A. punctulata* sperm was determined using the “pH_i_ pseudo-null-point” method (100–103). The method is described in refs. 28 and 44.

### Measurement of Sperm Motility.

Sperm motility was recorded under dark-field illumination using an inverted microscope with an EMCCD camera at 25 Hz (sea urchin) or 40 Hz (salmon). *A. punctulata* sperm were activated by superfusion with ASW. Quantification of the number of activated cells at different time points was done using custom-made software in MATLAB (Mathworks).

For photorelease experiments, sperm in ASF were loaded with caged 7-diethylaminocoumarin-4-yl)methyladenosine-3′,5′-cyclic monophosphate (DEACM-caged cAMP) or caged 6, 7- Bis(carboxymethoxy)coumarin-4-yl]methyladenosine- 3′, 5′-cyclic monophosphate (BECMCM-caged cAMP) and exposed to 390-nm light to release cAMP. The techniques using caged compounds are described in ref. 28. The percentage of cAMP released was calculated from photochemical properties of DEACM-caged cAMP (104) and the light intensity.

Motility was quantified using MATLAB-based image binarization and frame subtraction to generate a motility score, which was normalized to sperm density (mean pixel intensity) to correct for sample variation. Further details are provided in the *SI Appendix*.

### Statistical Analysis.

Statistical analyses of data from Figs. 2 and 4 were performed with scipy (version 1.15.1) and statsmodels (version 0.14.4) in python (3.12.3). The results of all analyses, including *P* values, are included in the Datasets published with this manuscript. **** indicates *P* ≤ 0.0001, *** indicates *P* ≤ 0.001, ** indicates *P* ≤ 0.01, * indicates *P* ≤ 0.05, and ns indicates *P* > 0.05.

## Supplementary Material

Appendix 01 (PDF)

Dataset S01 (XLSX)

Dataset S02 (XLSX)

Dataset S03 (XLSX)

Movie S1. Initiation of motility of quiescent sperm from *Arbacia punctulata* loaded with DEACM-caged cAMP after a flash of UV light that releases cAMP inside the cell.

Movie S2. Initiation of motility of quiescent sperm from *Salmo salar* loaded with DEACM-caged cAMP after a flash of UV light that releases cAMP inside the cell.

## Data Availability

Study data are included in the article and/or supporting information. It is also available in the repository https://edmond.mpg.de/dataset.xhtml?persistentId=doi:10.17617/3.5TFQ10 (105).
