# Supplementary material for: Soluble adenylyl cyclase in nonmammalian sperm is directly controlled by pH, not by HCO3− or Ca2+
Source: Proc Natl Acad Sci U S A. 2026 Jan 27;123(5):e2505026123. doi: 10.1073/pnas.2505026123 (PMC12867704; doi:10.1073/pnas.2505026123)
Supplement: Supplementary file 1 — Appendix 01 (PDF) [file pnas.2505026123.sapp.pdf]

Soluble adenylyl cyclase (sAC) in nonmammalian sperm is directly controlled by pH, not by HCO_3^- or Ca^{2+}.

Olivia Kendall[1,2], Oanh Tu Hoang[3], Joshua L. Wort[2], Hussein Hamzeh[1,4], Heinz G. Körschen[1], René Pascal[1], Kai Korsching[1], Meritxell Wu-Lu,[3] Wolfgang Bönigk[1], Christian Kambach[5], Luis Alvarez[1,6], Reinhard Seifert[1,4], Timo Strünker[1,4,7], Maria Andrea Mroginski[3], and U. Benjamin Kaupp[1,2,4,8]

[1]Max Planck Institute for Neurobiology of Behavior – caesar, Molecular Sensory Systems Group, Bonn 53175, Germany.

[2]Life & Medical Sciences Institute (LIMES), Molecular Sensory Systems, University of Bonn, 53115 Bonn, Germany

[3]Department of Chemistry, Faculty II, Technical University Berlin, Berlin 10623, Germany

[4]Marine Biological Laboratory, Woods Hole, MA 02543.

[5]Department of Biochemistry, University of Bayreuth, 95440 Bayreuth, Germany;

[6]Max Planck Institute for Neurobiology of Behavior – caesar, Neural Information Flow Group, 53175 Bonn, Germany.

[7]Center of Reproductive Medicine and Andrology, Molecular Reproductive Physiology, University Hospital Münster, University of Münster, 48149 Münster, Germany.

[8]Max-Planck-Institute for Multidisciplinary Sciences, Biophysics of Cellular Signal Transduction, 37077 Göttingen, Germany

Send correspondence to:

U. Benjamin Kaupp
Max Planck Institute for Multidisciplinary Sciences
Biophysics of Cellular Signal Transduction
Am Faßberg 11
37077 Göttingen, Germany
Tel.: ++49228-73-3830
e-mail: benjamin.kaupp@mpinat.mpg.de

O. Kendall
University of Bonn, LIMES Institute
Carl-Troll-Str. 31
53115 Bonn, Germany
Email: olivia.kendall@uni-bonn.de

Supplementary Figures

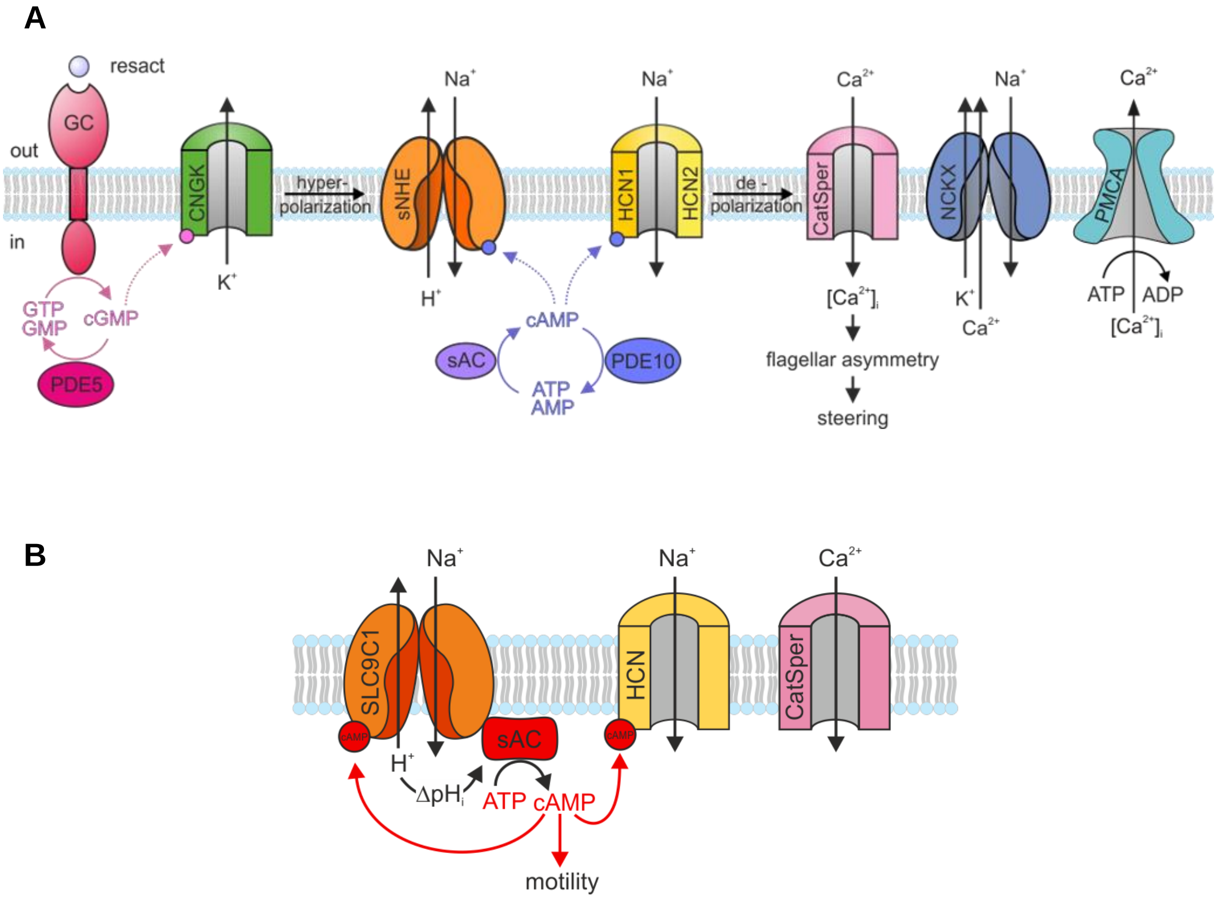

Supplementary Figure 1. (A) Chemotaxis signaling pathway in sea urchin sperm.

GC, receptor guanylate cyclase; CNGK, cyclic nucleotide-gated K^+-selective channel; sNHE/SLC9C1, sperm-specific Na^+/H^+ exchanger; HCN1, HCN2, hyperpolarization-activated, and cyclic nucleotide-gated channels; CatSper, cation channel of sperm; NCKX, Na^+/Ca^{2+}-K^+ exchanger; PMCA, plasma membrane Ca^{2+}-ATPase; PDE5, phosphodiesterase type 5; PDE10, phosphodiesterase type 10; sAC, soluble adenylate cyclase. For clarity, sAC is placed in the cytosol, although it is associated with the membrane; the same may apply to PDE10. From Trötschel et al. 2020. **(B) Model of the reciprocal control of sAC and SLC9C1.** The Na^+/H^+ exchanger SLC9C1 and the sAC reciprocally control each other's activity. cAMP controls three targets. First, it enhances the open probability of HCN channels and, thereby, contributes to CatSper channel opening during recovery from chemoattractant-evoked hyperpolarization; second, cAMP shifts the voltage dependence of SLC9C1 to less negative V_m; finally, cAMP controls dynein motor proteins via PKA-mediated phosphorylation (1, 2).

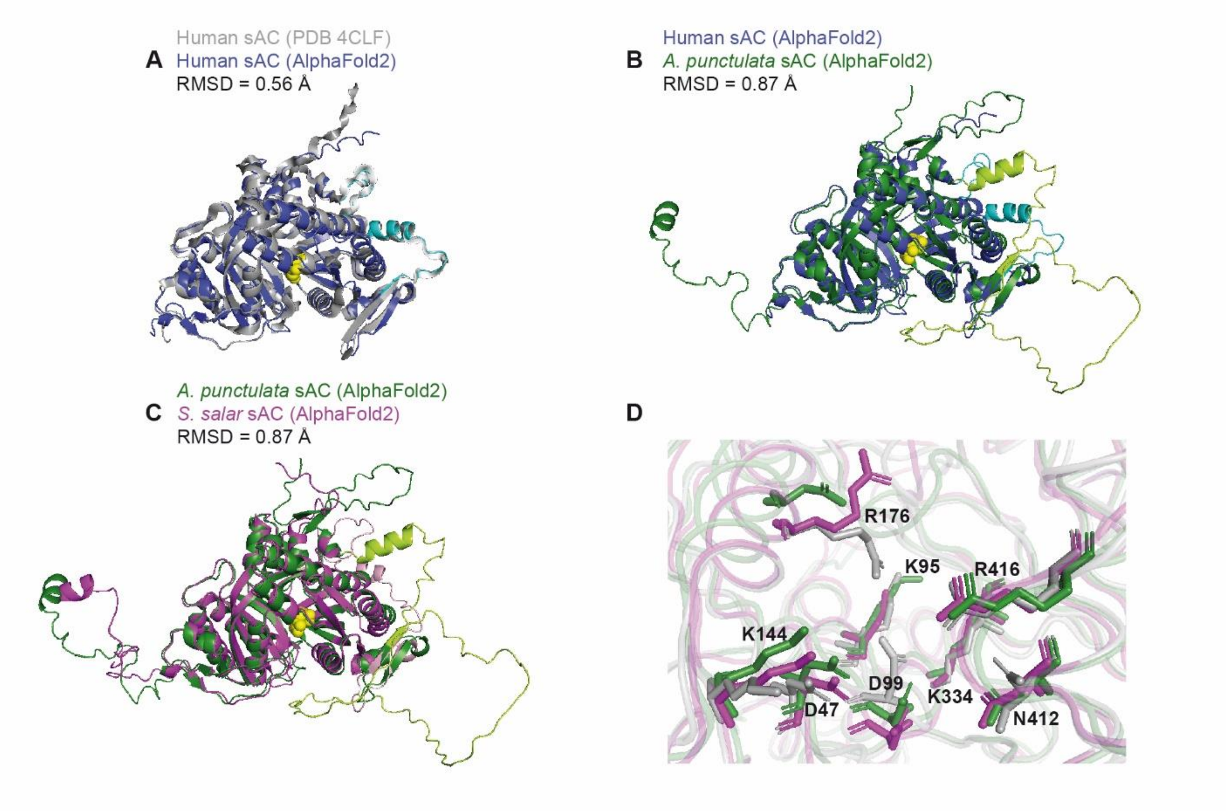

Supplementary Figure 2. Overlays of different models of soluble adenylyl cyclase (sAC). (**A**) Superposed structures of human sAC in apo-state (PDB: 4CLF) from X-ray crystallography (grey) and AlphaFold2 (blue), residue D99 is shown in yellow sphere representation as a reference point and proxy for HCO_3^-. The linker of human sAC from crystal structure and AlphaFold2 are shown in white and cyan, respectively. (**B**) Superposed structures of *Hs*sAC$_t$ (blue) and *Ap*sAC$_t$ (green), from AlphaFold2, residue D99 is shown in yellow sphere representation as a reference point and proxy for HCO_3^-. The linkers of human sAC (AlphaFold2) and *Ap*sAC$_t$ are highlighted in cyan and light green, respectively. (**C**) Superposed structures of *Ap*sAC$_t$ (green) and *Ss*sAC$_t$ (magenta), from AlphaFold2, residue D121 is shown in yellow sphere representation as a reference point and proxy for HCO_3^-. The linkers of *Ap*sAC$_t$ and *Ss*sAC$_t$ are highlighted in light green and light pink, respectively. (**D**) Residues involved in HCO_3^- binding and catalytic mechanism in *Hs*sAC$_t$ (PDB: 4CLF, grey), overlaid with *Ap*sAC$_t$ (green) and *Ss*sAC$_t$ (magenta) AlphaFold2 models. Residues are labelled according to *Hs*sAC$_t$ sequence and shown in stick representation.

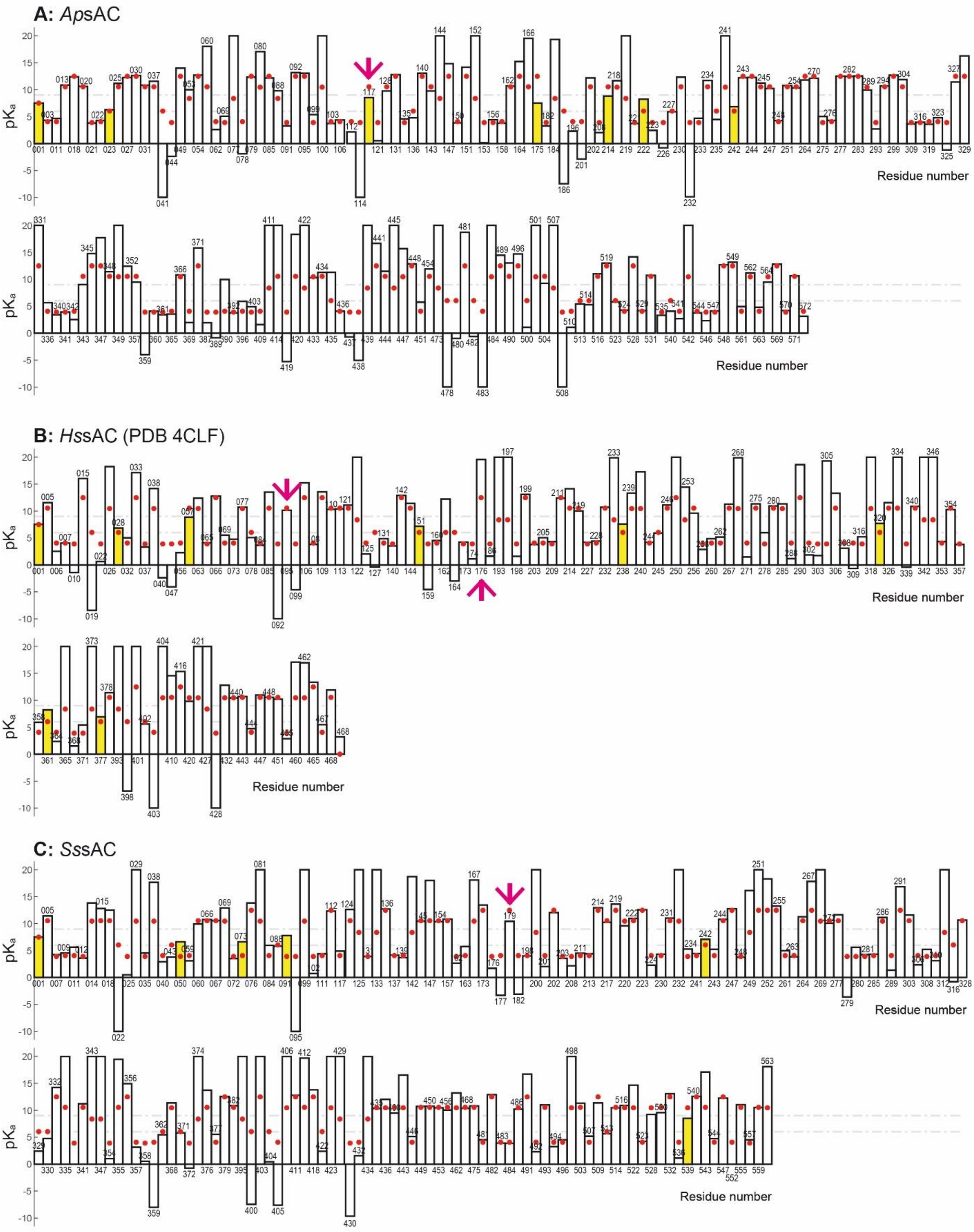
A: ApsAC
pKa
Residue number
B: HssAC (PDB 4CLF)
pKa
Residue number
C: SssAC
pKa
Residue number

Supplementary Figure 3. Karlsberg2^{+} titration of *Ap*sAC AlphaFold2 model (A)**, *Hs*sAC apo crystal structure (B), and *Ss*sAC AlphaFold2 model (C).** pK_a values are shown for residues with titratable side groups; this includes the N-terminus ("1") and all Asp, Cys, Glu, His, Lys, Arg and Tyr residues. The pK_a of the corresponding residue in aqueous solution is shown by red dots. The pK_a range 6-9 is indicated by two grey dotted lines, residues with an estimated pK_a that falls within this range are highlighted yellow. Residues that correspond to the bicarbonate-binding residues in *Hs*sAC are indicated by pink arrow.

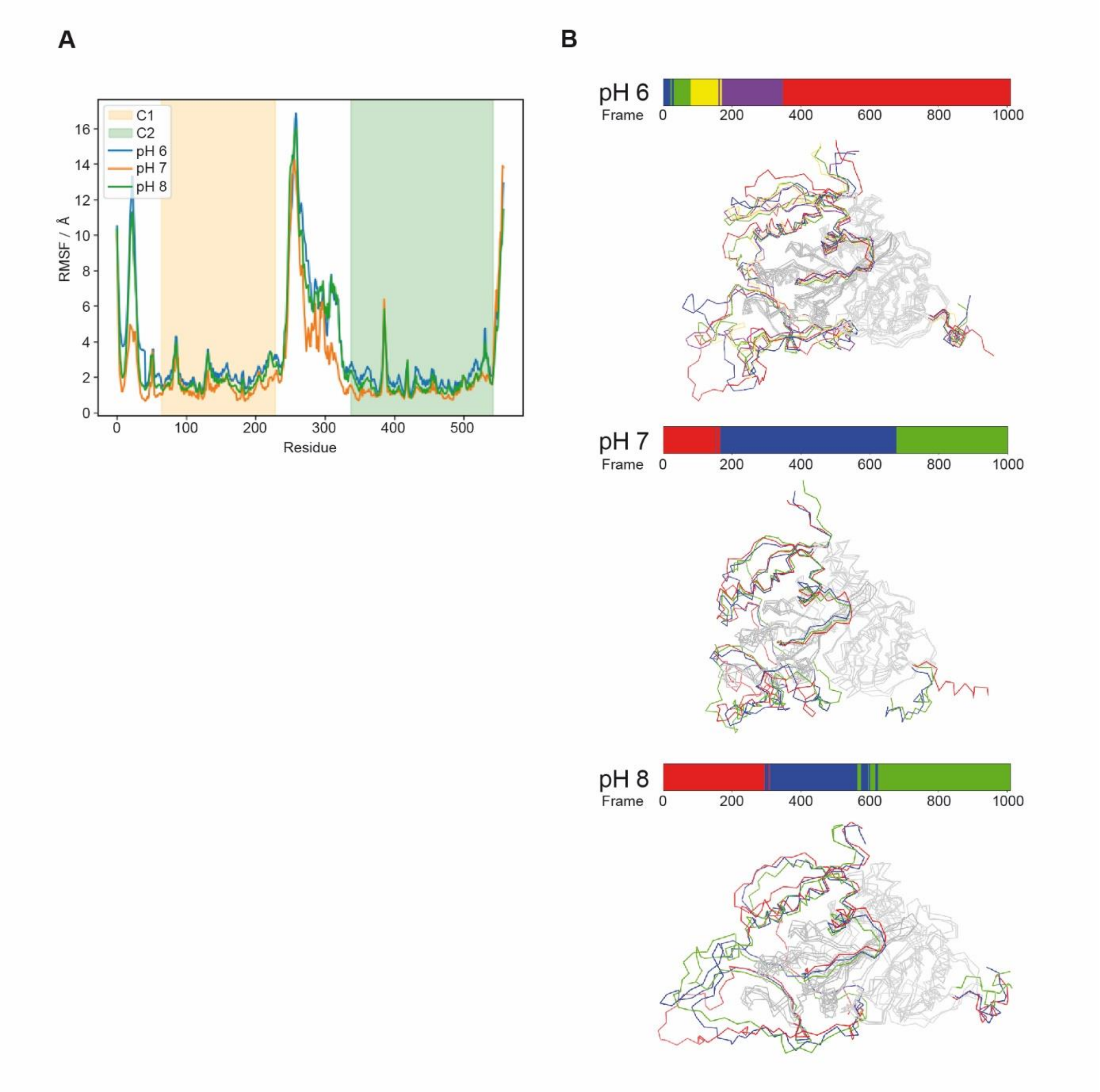

Supplementary Figure 4. Constant pH MD of *Aps*AC$_t$. (**A**) Root-mean-square fluctuation (RMSF) of constant pH MD simulations of *Ap*sAC$_t$ at pH 6 (blue), 7 (orange), and 8 (green), compared to the *Ap*sAC$_t$ AlphaFold model after equilibration. Residue numbers corresponding to catalytic domain 1 (C1, orange) and 2 (C2, green) are highlighted for reference. (**B**) TTclust analysis of one simulation each at pH 6, 7, and 8. The frames were clustered into similar conformations according to the RMSD of C-alpha atoms. The bar shows the frame at which the conformation was present; the model shows the corresponding conformation in ribbon form. For clarity, the C1 and C2 domains are colored grey, the linker is colored according to the conformation present.

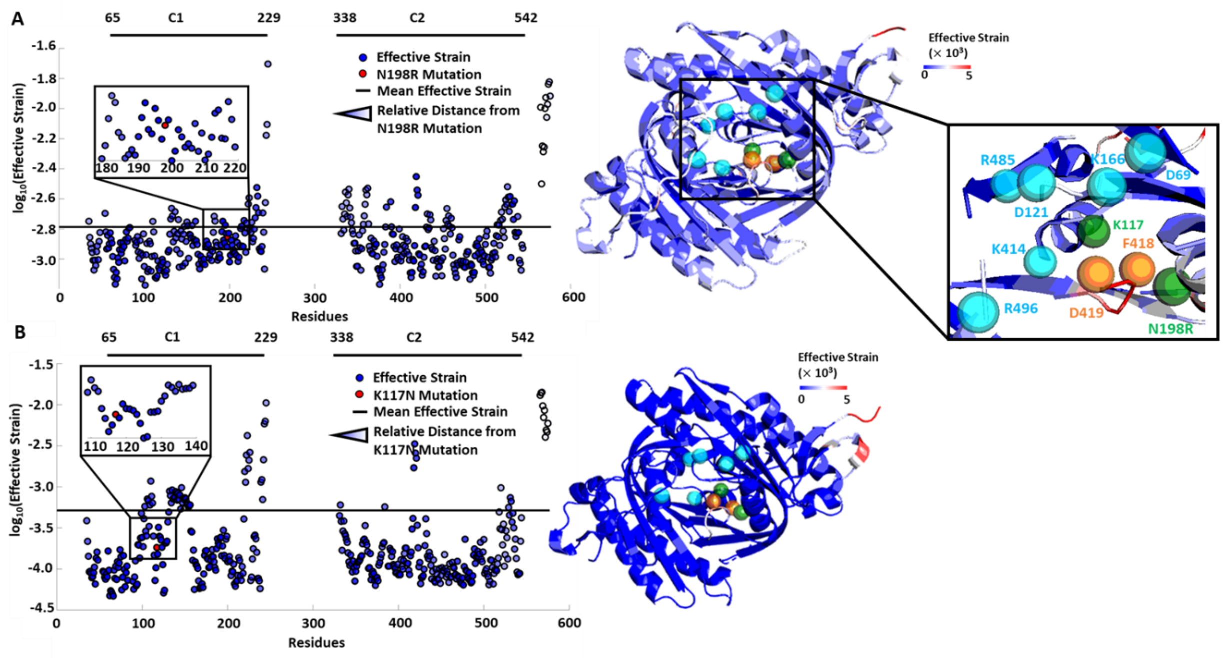

Supplementary Figure 5. Effective strain analysis of *A. punctulata* sAC mutants. (**A, B**; left) Scatter plots (blue) of log10(effective strain) calculated for the Cα atom of each residue (averaged over five AlphaFold2 structures, respectively). The mean effective strains over all included residues are indicated by black lines, and transparency of scatter points indicates relative distance of the residue from the mutation. Regions corresponding to residues in the C1 and C2 domains are indicated. Residues with pLDDT scores less than 70 were removed from further analysis. (inset) The region containing the mutation (scatter colored according to effective strain) to aid visualization. (**A, B**; right) Structures of *A. punctulata* colored blue-white-red by increasing effective strain (x 1000). Colored transparent spheres indicate residues listed in Table 1, cyan spheres indicate residues coordinate the ATP moiety, while green spheres indicate key residues involved in HCO_3^- coordination and sensing (in human sAC), and orange spheres indicate residues that either indirectly sense HCO_3^- binding, or interact with R176 (in human sAC). (inset). A close-up of the ATP- and HCO_3^--binding pocket. Residues of importance as listed in Table 1 are labelled.

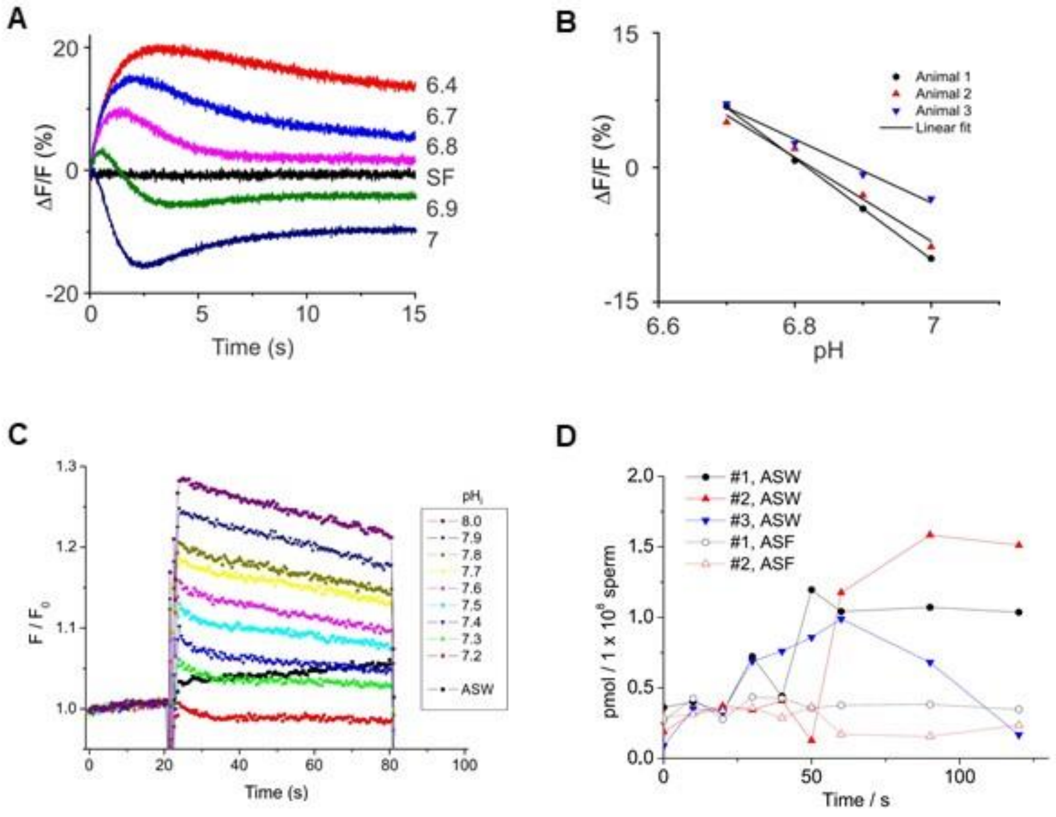

Supplementary Figure 6: Determination of pHi of *A. punctulata* sperm by the pH-clamp null-point technique, and time course of cAMP increase in *A. punctulata* sperm.

(**A**) Changes in relative fluorescence ΔF/F (%) after mixing sperm with pH-clamp solutions in artificial seminal fluid (ASF). (**B**) Plot of ΔF/F (%) *versus* pH-clamp value for three animals. The mean pH value at ΔF/F = 0 was 6.84 ± 0.02 (n = 3). **(C)** Changes in intracellular pH_i of *A. punctulata* sperm (3 x 10^8 cells ml^{-1}) after addition of different pH-clamp solutions at t = 20 s as indicated by color code. Changes in pH_i were measured in a Fluostar device with the pH indicator dye BCECF (10 µM); λ_{exc} = 485 nm, λ_{em} = 520 nm. **D)** Time course of cAMP increase after dilution by ASW of sperm incubated in ASF for 3 bioreplicates (solid lines, filled symbols; black, red, and blue). The individual time courses vary. This likely reflects damped oscillations caused by negative feedback of cAMP on sAC activity and cAMP hydrolysis by PDE due to the absence of IBMX, a PDE inhibitor. This is reminiscent of an internal Ca^{2+} oscillator, proposed by Ramirez-Gomez et al. (2020), and which is reflected in rapid Ca^{2+} signals evoked by caged cGMP (see Fig. 4e in Kaupp et al. 2003). It is not advisable to average data due to variations in the time course of individual oscillations, and selected time points in quenched-flow experiments may probe the oscillations at various stages of the oscillation. A control experiment of sperm diluted into ASF is also shown (solid

lines, open symbols). The cAMP values determined at t = 0 from all five measurements are given in the main text as resting cAMP levels.

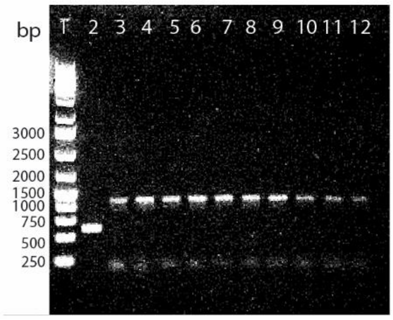

Supplementary Figure 7: Mycoplasma tests of HEK cell lines.

Mycoplasma test of HEK cell cultures using the PCR mycoplasma test kit I/C (PromoKine GmbH). The layout is as follows: 1, GeneRuler 1 kb; 2, positive control from manufacturer; 3, negative control (M10 media); 4, CHO; 5 and 6, HEK293; 7 and 8, HEK ATCC; 9, HEK TM; 10, HEK-*Ap*sAC$_{fl}$; 11, HEK-*Ap*sAC$_{t}$; 12, HEK-*Ss*sAC$_{t}$.

Supplementary Movie 1: Initiation of motility of quiescent sperm from *Arbacia punctulata* loaded with DEACM-caged cAMP after a flash of UV light that releases cAMP inside the cell.

Supplementary Movie 2: Initiation of motility of quiescent sperm from *Salmo salar* loaded with DEACM-caged cAMP after a flash of UV light that releases cAMP inside the cell.

SI Appendix

Methods

Bioinformatics

Genomes available from NCBI [https://www.ncbi.nlm.nih.gov/genome/] as of November 2021. The search was carried out in the default database for blastp and tblastn (non-redundant protein sequences (nr) for blastp, nucleotide collection (nr/nt) for tblastn). For tblastn, some searches were also performed in whole-genome shotgun contigs (wgs). As query sequence, we used *Homo sapiens* sAC or additional annotated sACs that were more closely related to the target group. The search was restricted to the groups listed in Supplementary Table 2. For searches in wgs, candidate proteins were predicted using *genewise* [https://www.ebi.ac.uk/Tools/psa/genewise/] (3) and the query sequence used for tblastn. Candidates were tested using phylogenetic tree analysis including (i) other sAC proteins from that group, (ii) a standard list of sAC orthologs (human, rat, coelacanth, and lungfish), and (iii) as outgroup, members of the family of membrane-spanning adenylate cyclases (tmAC). To construct the tree, sequences were first aligned using the MAFFT tool version 7 [https://mafft.cbrc.jp/alignment/server/] (4, 5). The strategy parameter was set to E-INS-I, otherwise default parameters were used. The alignment was stripped of gaps using Gap strip/squeeze v2.1.0. [https://hcv.lanl.gov/content/sequence/GAPSTREEZE/gap.html]. The gap tolerance threshold was set to 80%. The tree was calculated with the PhyML online tool [http://www.atgc-montpellier.fr/phyml/] (Guindon *et al*, 2010), with the tree improvement method set to SPR, and the branch support set to chi-square-based aLRT. Candidate proteins, which fall with a high branch support (> 90%) into a sAC clade, were considered validated. Validated sAC proteins, along with previously annotated sAC, were aligned with *Homo sapiens* sAC using the MAFFT tool. The alignment was stripped of gaps using Gap strip/squeeze v2.1.0 with a gap tolerance of 50-60%. The sites of polymorphism were located using the *Homo sapiens* sequence as reference.

Computational Methods - AlphaFold2 modeling, effective strain analysis, and MD simulations of pK_A values

A homology model of *Ap*sAC was generated with Modeller v9 (6), using the closest homologs from the PDB as templates: a cyanobacterial adenylate cyclase from the *S. platensis* (PDB ID 1WC0) (7) for C1 and human sAC-C2 (PDB ID 4CLL) (8) for C2. The pK_a of residues in the homology model were predicted with Karlsberg2$^+$ (9).

Modelling in AlphaFold2 v2.2.4 was performed within the ColabFold v1.5.2 pipeline (10), with multiple sequence alignments generated using MMSeqs2 (11) and without the use of template information. All parameters were set to default (auto) and AMBER relaxation was not performed. The five output unrelaxed structures were ranked by the predicted local distance difference test (pLDDT) scores, with the structures corresponding to the highest pLDDT score shown in the main text and Supplementary Figure 2.

Constant pH MD simulations were performed with AMBER 22 with the Amber ff14SB force field (12, 13). The AlphaFold model of *Ap*sAC$_t$ was used as a starting template. D69, K117, D121, K214, D419 were chosen to be titrated, as well as all histidine residues (20 total). Protonation states of the remaining Asp, Glu, and Lys residues were assigned according to the Karlsberg2$^+$ prediction. The system was solvated in a box of 20 Å TIP3P solvent buffer between the edges of the box and the protein. Then, the box was neutralized with Na^+ ions with tleap. All simulations were performed with 2 fs time steps, with a 12 Å cut off for van der Waals interactions, and bonds containing hydrogen were constrained with SHAKE. The system was minimized using 10,000 steps of the steepest descent method. The minimized structure was heated stepwise from 100 K for the first 450,000 steps to 300 K with Langevin dynamics for 200 ps and restrained backbone atoms with 1.0 kcal/mol Å^2. The heated structure was then equilibrated using Langevin dynamics with a friction coefficient of 10 ps^{-1} for 200 ps at 300 K. After, a production run was performed for 100 ns for each pH (6, 7, and

8), attempting to change the protonation every 100 steps. During the constant pH production run, periodic boundary conditions for constant volume were used with an anisotropic pressure control and a Langevin thermostat collision frequency of 2.0. Twenty replicates were calculated for each pH value. The trajectories were analysed with ttclust (14), cphstats, and AmberTools22 (13).

For the effective strain analysis of the N198 and K117N mutations, the AlphaFold model of wild-type *Ap*sAC$_{fl}$ provided a 'control' structure for pairwise comparison with structures containing either mutation. The effective strain metric is comparable to local root-mean square deviations (within neighbourhoods of defined radius; 13 Å in this example) of Cα atoms and that are normalized by the distance from the mutation site. To improve robustness and reproducibility, effective strain was calculated and averaged for five AlphaFold2 structures, and regions with a pLDDT score of less than 70 were excluded from further analysis. The MATLAB code used for this analysis is available as an additional Supplementary File (Effective Strain Analysis code).

Cloning of full-length and truncated sAC and sAC mutants

Three sets of primer pairs, designed using genomic data of *A. punctulata*, were used to obtain the full-length sequence of soluble adenylate cyclase (sAC) by PCR amplification on an *A. punctulata* testis library. Primers C3299 and C3302 were used to amplify the 5′ part of the sequence (bp 1 to 1790). Primer C3299 introduces a BamHI site followed by a perfect Kozak sequence preceding the start codon. Primer C3302 introduces an EcoRI site by silent mutation. Primers C3307 and C3310 were used to amplify bases 1768 to 3717. Primer C3310 introduces a XbaI site by silent mutation. The 3′ part (bp 3704 to 5556) was amplified with primers C3315 and C3318, followed by a PCR with primers C3315 and C1001, which adds a sequence for an HA-tag to the 3' end followed by a stop codon and a NotI site. The primer sequences were:

CACCGGATCCACCATGAGTGAGGCTATAAACTCAACACAG (C3299)

ACCAGAATTCTCCGATGGTCTCCTCTCTCTCGC (C3302)

GGAGACCATCGGAGAATTCTGGTGTTCCAGGG (C3307)

GCCTTCTAGAGGTTCATCATCATCTGAGGAATC (C3310)

AACCTCTAGAAGGCAGACCCAAGGAATATAAC (C3315)

TAATAAGCGGCCGCTAGGCGTAGTCGGGCACGTCGTAGGGGTATTCCTCTTCACC
CCTGGGGAGAG (C3318)

TAATAAGCGGCCGCTAGGCGTAGTCGGGCACGTCGTAGGGG (C1001)

The three resulting PCR fragments were cloned into vector pcDNA6/V5-HisA (Invitrogen, Carlsbad, USA) digested with BamHI and NotI to obtain the full-length clone. The DNA for the *S. salar* sAC was synthesized according to the annotated sequence for the soluble adenylate cyclase 10, XM_014151728. A HA-tag was added to the C-terminal end. The DNA was cloned via HindIII and XbaI sites into vector pcDNA3.1/zeo(+) (Invitrogen, Carlsbad, USA).

Truncated sAC from *Arbacia punctulata* (Met 1 to Leu 577) was constructed by successive PCRs using 5' primer C3299 and 3' primers C3680, C3681, C3682. Primer C3680 matches the *Ap*sAC gene up to L577 and adds a part of the HA-tag, primer C3681 adds part of the HA-tag, followed by a short linker (Gly, Ser, Gly), primer C3682 adds a hexa His-tag, followed by a stop codon and a XbaI site. The resulting PCR fragment was cloned into vector pcDNA6/V5-HisA (Invitrogen, Carlsbad, USA). The primer sequences were:

CACCGGATCCACCATGAGTGAGGCTATAAACTCAACACAG (C3299)
GGGCACGTCGTAGGGGTACAAGAAGATAGACATCTCCTTGTCTCG (C3680)
GGTGGCCGCTGCCGGCGTAGTCGGGCACGTCGTAGGGGTACAAG (C3681)
ATATCTAGATTAGTGGTGGTGGTGGTGGTGGCCGCTGCCGGCGTAG (C3682)

Truncated sAC from *Salmon salar* (Met 1 to Ser 499) was constructed by successively PCRs using 5' primer C3978 and 3' primers C4256, C4257, C3682. Primer C4256 matches the

*Ss*sAC gene up to S499 and adds a part of the HA-tag, primer C4257 adds part of the HA-tag, followed by a short linker (Gly, Ser, Gly), primer C3682 adds a hexa His-tag, followed by a stop codon and a XbaI site. The resulting PCR fragment was cloned into vector pcDNA6/V5-HisA (Invitrogen, Carlsbad, USA). The primer sequences were:

TAAGCTTCCACCATGGGCTGGATCAAGGGCGACGGCGAGATCGAG (C3978)
GTCGGGCACGTCGTAGGGGTAGCTGTACACCTCGATCTCCTTCTCC (C4256)
GTGGTGGCCGCTGCCGGCGTAGTCGGGCACGTCGTAGGGGTAGC (C4257)
ATATCTAGATTAGTGGTGGTGGTGGTGGTGGCCGCTGCCGGCGTAG (C3682)

Truncated sAC from *R. norvegicus* with a HA-tag added to the C-terminal end representing the N-terminal domain (Met 1 to Val 469) of sAC (Buck *et al*, 1999) was cloned into pcDNA5/FRT (Invitrogen).

Cell lines

For heterologous expression, HEK293 cells from the European Collection of Authenticated Cell Cultures (ECACC) were used. Mycoplasma tests were carried out regularly once a year using the PCR mycoplasma test kit I/C (PromoKine GmbH), according to manufacturer instructions (Supplementary Figure 7).

Generation of HEK293 cell lines stably expressing *Ap*sAC$_t$, *Ss*sAC$_t$, or *Rn*sAC$_t$

HEK293 cells were electroporated with plasmids using the Neon 100 Kit (Invitrogen, Carlsbad, USA) and a MicroPorator (Digital Bio) according to the manufacturer's protocol (3 × 1245 mV pulses with a 10-ms pulse width). Cells were transferred into complete medium composed of M10 plus GlutaMAX or DH10 plus GlutaMax (Invitrogen) and 10% fetal bovine serum (Biochrom, Berlin, Germany). To select monoclonal cells stably expressing sAC, the respective antibiotic was added to the cell culture medium 24 h after electroporation. Monoclonal cell lines were identified by immunocytochemistry using a rat anti-HA antibody (Roche Applied Science). HEK293 cells stably overexpressing *Rn*sAC$_t$ were as described (15).

Expression and purification of the *Ap*sAC$_t$ catalytic core

The PCR fragment *A. punctulata* sAC (aa 1-577) with a C-terminal HA and His tag, *Ap*sAC(1-577)HAH, was cloned into a pET21a vector. This construct served as the template for all mutants that were made using the QuikChange Protocol (16) with primers detailed in Supplementary Table 6. The *E. coli* strain BL21(DE3)pLysS were transformed with pET-*Ap*sAC(1-577)HAH and grown in LB media to OD_{600} = 0.7. After IPTG induction (0.1 mM) for 6 hours at 19 ^oC, the cells were harvested and stored until purification at -20 ^oC. All purification steps proceeded at 4 ^oC or on ice. The cells were resuspended in sAC buffer (50 mM HEPES pH 8.0, 250 mM NaCl, 10% Glycerol, 0.3 mM DDM, 1 mM DTT) supplemented with 1 mM DDM, 0.5 mM PMSF, Pierce protease inhibitor tablet, EDTA-free). The suspension was lysed by sonication. The clarified lysate was applied to a 1 mL HiTRAP TALON crude column, washed with sAC buffer containing 15 mM imidazole, then eluted with sAC buffer containing 250 mM imidazole. The protein was buffer-exchanged with a PD10 column to sAC buffer and supplemented with Halt Protease inhibitor cocktail with 5 mM EDTA. The protein was concentrated with Vivaspin spin concentrators, then applied to Superdex Increase 75 pg 10/300, equilibrated with sAC buffer excluding glycerol. The purified protein was either used immediately for activity assays or stored with 10% glycerol at -80 ^oC.

The purity and concentration of purified *Ap*sAC$_t$ was determined by comparing the extent of Coomassie staining to a BSA standard on an SDS PAGE gel. A 0.1 mg/mL BSA standard was prepared in sAC buffer with 8 × Laemmli Buffer (125 mM TrisHCl pH 6.8, 20% glycerol, 4% SDS, 10% beta-mercaptoethanol, bromophenol blue), and stored in aliquots at -20 ^oC. The total protein concentration of the sAC sample was measured by Nanodrop (1 Abs = 1 mg/mL) and a protein sample with estimated 0.1 mg/mL was prepared with 8 × Laemmli Buffer. All samples were boiled at 95 °C for 10 min and spun 2 min 17,000 x g at room temperature, then applied to a 12% SDS PAGE gel. The gel was run for 60 min at 30 mA using the Biometra

Minigel Twin system. The gel was fixed in fixing solution (50 % MeOH, 10 % Acetic acid) for 10 min at 180 rpm, stained with Coomassie Dye (0.25 % in 45 % MeOH, 10 % acetic acid), then destained in fixing solution, 2 x 20 min, and MilliQ, 2 x 20 min. The gel was imaged in a Vilber Lourmat E-BOX chamber by UV and white light illumination. The intensity of the *Ap*sAC$_t$ and BSA bands were calculated with ImageJ 1.54f, by fitting a Gaussian curve to the line profile of the band at 65 kDa (the band corresponding to *Ap*sAC$_t$). The concentration of *Ap*sAC$_t$ was calculated from the area under the curve with a custom-made python script (Python 3.11.9).

Measurement of sAC activity and quantification of cAMP content in sperm and cell lines

The cAMP synthesis of intact sperm from sea urchin and salmon under various salt and pH conditions on the sub-second time scale was analyzed using the stopped-flow instrument. Experiments on the seconds to minutes time scale were performed at room temperature in 96-well Protein LoBind plates (Eppendorf, Hamburg, Germany).

The cAMP synthesis of heterologously expressed sAC in HEK293 lysates was induced by applying di-sodium-ATP (Sigma Aldrich) and $MgCl_2$ or $MnCl_2$ in isotonic HEPES buffer. If not otherwise stated, reaction buffer contained (in mM): 1 ATP, 5 $MgCl_2$ or 5 $MnCl_2$, 0.3 $CaCl_2$, 120 KCl, 12 NaCl, 0.2 IBMX, 50 HEPES/KOH (at pH 7 or pH 8), and optional 25 HCO_3. Reaction time was 10 minutes for assays with HEK293 lysates. Reactions were stopped by quenching with 0.5 × reaction volume $HClO_4$ (1.5 M). The solution was neutralized with 0.625 × reaction volume K_3PO_4 (1 M). Salt precipitate and cell debris were removed by centrifugation of the 96-well plates (10 min, 4,000×g, 4 °C). The supernatant was transferred into a new 96-well plate.

The cAMP synthesis of purified *Ap*sAC$_t$ was induced by applying di-sodium-ATP (Sigma Aldrich) and $MgCl_2$ or $MnCl_2$ in isotonic HEPES buffer. If not otherwise stated, reaction

buffer contained (in mM): 1 ATP, 5 $MgCl_2$ or 5 $MnCl_2$, 120 KCl, 12 NaCl, 50 HEPES/KOH, 0.1 $CaCl_2$, and 0.6 BAPTA (resulting in $[Ca^{2+}]_{free}$ = 50 nM). When testing the Ca^{2+} dependence of ApsACt (Figure 2E and F), BAPTA was omitted from the reaction buffer. For assays containing 25 mM HCO_3^-, a more acidic HEPES stock was used to compensate for the alkalization caused by HCO_3^- addition, which arises from the chemical equilibrium $CO_2 + H_2O \rightleftharpoons HCO_3^- + H^+$. The final pH of the assays was confirmed using a Greisinger GMH 3530 pH electrode. Assays were quenched after 10 minutes by addition of 100 µL 12 mM EDTA, which reduced $[Mg^{2+}]_{free}$ to ca 20 µM or $[Mn^{2+}]_{free}$ to ca 0.2 nM, respectively.

The total content of cAMP in quenched samples was determined by using the CatchPoint cAMP Fluorescent Assay Kit (Molecular Devices, San Jose, USA) with some modifications, in particular to adjust reactions volumes to the 8- or 12-channel pipettes (Eppendorf, Hamburg, Germany), repetitive pipette HandyStep (Brand, Wertheim, Germany), or the Multi-Channel Auto Sampling System NSP-7000 (Nichiryo, Japan). Briefly, 60 µl of the quenched sample and 50 µl each of the anti-cAMP antibody and the HRP-cAMP conjugate were applied to an anti-rabbit IgG-coated 96-well plate according to the manufacturer’s protocol. After 90 min of incubation at room temperature and repetitive washing (3 times) with wash buffer (125 µl each), the Stoplight Red Substrate (125 µl) was applied to monitor the immunoreaction. The 96-well plates were analyzed using a FLUOstar Omega microplate reader (BMGLabtech, Ortenberg, Germany). The amount of cAMP was quantified using calibration curves obtained by serial dilutions of cAMP standards. Analysis was done using the reader’s data analysis software MARS. Further data processing was done using Excel (Microsoft, USA) OriginPro (Origin lab, USA), and Python 3.12.4, using the packages wellmap (17), and matplotlib 3.9.1. Figures were prepared using Adobe Illustrator v. 15.0.3.

The cAMP content of intact HEK293 cells stably expressing *Rn*sAC$_t$ (2.0 x 10^6 cells) was determined by an accumulation assay (15). Cells were transferred to 1.5 ml tubes and

incubated in DMEM + 10% FBS in suspension at 37ºC, 5% CO_2 for one hour. A time zero value for each condition was determined by adding 100 µl of cells directly into 100 µl stop solution (0.2 M HCl). To measure cAMP accumulation, cells in suspension were incubated for the indicated period of time in the presence of 500 µM IBMX at 37ºC after which 100 µl of cells were transferred to a fresh tube containing stop solution. Intracellular cAMP content was determined using Correlate-EIA Direct Assay (Assay Designs, Inc).

Some general complications of pH control when working with HCO_3^- are described in the Discussion. Additional complications arise when interpreting experiments using HCO_3^- and pH in intact cells or cell lysates. Because of energetics, membrane fragments in cell lysates form vesicles of various sizes. The vesicles can be categorised in two configurations: inside-out, where the cytoplasmic side of the membrane forms the outside of the vesicle, or outside-out, where the extracellular side of the membrane forms the outside of the vesicle. As sAC strongly associates to the membrane (18–21), the sAC may be either inside or outside of the vesicles, and thus will be exposed to different conditions when HCO_3^- or buffers with different pH are added. Furthermore, the vesicles may contain varying numbers of carbonic anhydrases and HCO_3^- transporters, which will alter the HCO_3^- levels inside the vesicle. Altogether, these factors contribute to a heterogeneous population of sAC molecules, exposed to different pH levels and HCO_3^- concentrations. Therefore, the cleanest way to determine the HCO_3^-/pH sensitivity of a protein is to work with purified protein, where the reaction mixture can be precisely defined and controlled.

Simulation of spawning in the stopped-flow device, and measurement of changes in V_m and pH_i in *A. punctulata* sperm

To simulate spawning, we diluted sperm suspensions in the stopped-flow device and measured changes in pH_i and V_m using the respective fluorescent dyes (BCECF-AM or pHrodo Red-AM from Molecular Probes for pH, Eugene, USA, and FluoVolt or Di-8-

ANNEPS from Thermofisher for V_m). Dye-loading of sperm occurred at 18 ^oC by suspending dry sperm 1:6 (v/v) either in artificial seawater (ASW) or in artificial seminal fluid (ASF), which contained the respective dyes. ASW contained (in mM): NaCl 423, $CaCl_2$ 9.27, KCl 9, $MgCl_2$ 22.94, $MgSO_4$ 25.5, EDTA 0.1, HEPES 10 at pH 7.8. ASF was ASW containing 27 mM [K^+] at pH 6.7. Loading concentrations and times were: BCECF-AM: 10 µM for 10 min, no Pluronic; pHrodo Red-AM: 10 µM for 30-45 min (0.5% Pluronic F127); FluoVolt: 5 µM for 5 min, no Pluronic. After incubation, the sperm suspension was diluted 1:20 in ASF. This diluted sperm suspension was then rapidly mixed in a stopped-flow device (SFM-400, BioLogic, Claix, France) (22) with K^+- free ASW that was fortified with 50 mM Hepes at pH 7.8 (0KASW7.8). After 1:2 mixing, the [K^+] was 9-10 mM and pH was 7.7-7.8. As a control, we mixed sperm suspensions with 30KASW7.8; after mixing, the final [K^+] was 29 mM and the final pH_o was 7.7-7.8. Sperm suspensions and solutions were mixed in the stopped-flow device at flow rates of 2 ml s^{-1}.

Fluorescence was excited using pulsed LED light (SpectraX Light Engine, Lumencor, Beaverton, USA or M490L3, Thorlabs, Newton, USA) at a frequency of 10 kHz. The emitted fluorescence was captured by photo-multiplier modules (H9656-20 and C7169, Hamamatsu Photonics, Japan). The signal was amplified and filtered by a lock-in amplifier (7230 DualPhase, Ametek, Paoli, USA), and data acquisition was performed with a data acquisition pad (PCI-6221, National Instruments, Austin, USA) and Biokine Software v.4.49 (BioLogic). BCECF fluorescence was excited using a 452/45 nm filter (Semrock) and recorded in dual-emission mode using Brightline 494/20 nm and 540/15 nm filters (Semrock). The pH_i signals represent the ratio of F494/F540. All signals are the average of at least two recordings and are depicted as the percent change in ratio (ΔR/R) with respect to the first 10–20 data points after mixing. The pHrodo Red dye was excited at 572/15 nm and the emission was collected at 628/40 nm. Fluovolt was excited using a 513/18 nm filter (Semrock) and its emission collected at 540/20 nm (Semrock). Di-8-ANEPPS was excited at 475/20 nm (Semrock) and

its fluorescence was recorded in dual-emission mode using Brightline 536/40 nm and 628/40 nm filters (Semrock). The V_m signals (ratio 536/549 nm (R); average of at least four recordings) are depicted as the percent change in ratio ($\Delta R/R$) with respect to the first 10 data points. The signal recorded upon mixing sperm with ASF in the stopped-flow device represented the baseline control and was subtracted from the respective signals. The techniques are comprehensively described in (22).

The pH-clamp method

We determined the pH_i sensitivity of sAC in intact *A. punctulata* sperm using the "pH_i pseudo-null-point" method (23–26) that allows clamping of pH_i to fixed values and calibration of the pH indicator BCECF. The method for sea urchin sperm is described in (22, 27). Key is a set of pH_i-clamp solutions composed of a weak acid (butyric acid, BA) and a weak base (trimethylamine, TMA) at different molar ratios. TMA and BA freely equilibrate across the membrane and, at sufficiently high concentrations, establish a defined pH_i that is set by the acid/base ratio (24). The pH_i-null-point solutions were prepared according to the following equation: $pH_i\text{-null} = pH_o - 0.5 \log ([BA]/[TMA])$; pH_o = extracellular pH (7.8) (25), wherein [TMA] indicates the concentration of trimethylamine and [BA] that of butyric acid. The [BA] was 15 mM.

Measurement of sperm motility

Sperm motility was recorded in an inverted microscope (Olympus IX71) equipped with a 10x objective lens (UPlanSApo 10x, 0.4 NA; Olympus) under dark-field illumination at 25 Hz (sea urchin sperm) or 40 Hz (salmon sperm) with an EMCCD camera (DU-897D; Andor Technology). *Arbacia punctulata* sperm were diluted 1:200 in artificial seminal fluid (ASF) containing 0.5% Pluronic F127 (Sigma-Aldrich) and loaded into glass capillaries for superfusion (Rectangle Boro Tubing 0.2x4 mm; Vitro Tubes). The 150-mm loading tube (0.86 mm ID, 1.52 mm OD Fine Bore Polythene Tubing; Smiths medical) was then placed

into artificial seawater (ASW) with 0.5% Pluronic F127 to prevent sticking to the glass surface. Starting the perfusion results in mixing ASF with sperm and replacing the ASF with ASW. After dilution of the sperm suspension by 1:100 in ASW, the perfusion was stopped and the time course of activation was recorded. Quantification of the number of activated cells at different time points was done using custom-made software written in MATLAB (Mathworks, available in Supplementary Folder, SACY CASA). *Salmo salar* sperm (dilution 1:2770) or *Arbacia punctulata* sperm were loaded for five minutes in salmon or sea urchin ASF containing 4 µM caged 7-Diethylaminocoumarin-4-yl)methyladenosine-3',5'-cyclic monophosphate (DEACM-caged cAMP) and 0.5% Pluronic F127 (Sigma-Aldrich). In some experiments with *A. punctulata* sperm, in addition caged 6, 7- Bis(carboxymethoxy)coumarin-4 -yl]methyladenosine- 3', 5'- cyclic monophosphate (BECMCM-caged cAMP) was used. The techniques using caged compounds are described in (22).

For imaging, sperm were loaded into custom-made glass chambers with 150 µm height. A 390-nm light source (SpectraX Light Engine; Lumencor) was used to release cAMP. The power of the UV (147 mW maximal power) was measured using a power meter (Controller PowerMax and head model PS19Q; Coherent). The flash intensity was graded using neutral density filters (Absorptive ND Filters; Thorlabs), and the duration of the flash (5 ms length) was controlled using a custom-made LabVIEW software. The percentage of cAMP released was calculated based on the photochemical properties of DEACM-caged cAMP (28) and the light intensity at the focal plane of the microscope. The motility indicator was calculated using custom-made software written in MATLAB (Mathworks, available as additional Supplementary file, Sperm motility code). In brief, the software binarizes the images using an automatic threshold (graythres). Sperm motility can be coarsely quantified by subtracting temporally-adjacent frames. The absolute value of this image difference is then integrated over all pixels to produce a motility score. When sperm are perfectly static, such score becomes zero and gradually increases as cells move. The relative sperm density was

quantified as the mean pixel intensity of the binarized image. To compensate for slight variations in sperm density, this value was used to normalize the motility score.

Supplementary Table 1: Overview of papers reporting sAC regulation in various organisms

Paper	Organism	Main sAC regulation	Notes
Cook & Babcock, *J.Biol.Chem.*1993	Sea urchin (*S. purpuratus*)	pH	cAMP increase in sperm at alkaline pH_i
Beltran et al., *Biochemistry*, 1996	Sea urchin (*S. purpuratus, L. pictus)*	Membrane potential, pH	Alkalization with NH_4^+ stimulates sAC by 1.9xfold, larger than with HCO_3^-.
Nomura et al., *Gene,* 2005	Sea urchin (*S. purpuratus)*	pH	
Beltran et al., *Biochem. Biophys. Res. Commun.*, 2007	Sea urchin (*S. purpuratus)*	HCO_3^-	sAC also regulated by pH, G proteins, Ca^{2+}, PKA, hyperpolarization.
Vacquier et al., *BBA*, 2014	Sea urchin	pH/HCO_3^-	HCO_3^--binding residues from *Hs*sAC are conserved in sea urchins
Tresguerres et al., *PNAS*, 2010	Dogfish	HCO_3^-	slightly pH sensitive
Tresguerres et al., *J. of Exp. Biol.,* 2014	Aquatic organisms	HCO_3^-	Review
Barott et al., *Sci. Rep.*, 2013	Corals	HCO_3^-	sAC inhibited by Ca^{2+}
Barott et al., *Proc. Biol. Sci.*, 2017	Corals	HCO_3^-	sAC described as pH sensor<br>sAC modulates pH_i
Speer et al., *PNAS*, 2021	Corals	Unclear	cAMP increases due to alkalization. Reference made to both Nomura et al., 2005 (pH-sensitive) and Beltran et al., 2007 (HCO_3^--sensitive)
Salmeron et al., *Interface Focus*, 2021	Rainbow trout	HCO_3^-	

Discussion of Supplementary Table 1.

This table illustrates the conflicting conclusions about the regulation of sAC and cAMP of non-mammalian sperm in the literature. In the following, we discuss some of the papers and, in particular address two issues that we would like to comment on here:

1) the pH vs. HCO_3^- sensitivity of non-mammalian sAC (e.g., sea urchin, fish, corals);
2) the distinction between pH/HCO_3^--sensitive/pH/HCO_3^--dependent versus pH/HCO_3^--regulated enzyme.

pH vs. HCO_3^- sensitivity of sAC from sea urchin, fish, and corals. Long before the discovery of sAC in sperm, Cook & Babcock (1993) demonstrated that manipulations that increase sperm pH_i (e.g., addition of NH_4Cl) enhance cAMP levels and downstream signalling events (29). Beltran et al. (1996) also show that alkalization by NH_4Cl increased sAC activity by 1.9xfold

(30). Moreover, their finding that hyperpolarization stimulates cAMP synthesis is consistent with voltage-gated activation of Na^+/H^+ exchange by SLC9C1 followed by alkalization, which then stimulates sAC activity. Nomura et al. (2005) demonstrate direct pH sensitivity in recombinant sea urchin sAC (31). Vacquier et al. (2014) seem to be undecided regarding pH vs. HCO_3^- sensitivity (32). The authors refer to their previous report on the pH sensitivity of recombinant sea urchin sAC; at the same time, they emphasize that the HCO_3^--binding residues are strictly conserved in sea urchin sAC, suggesting that the sAC can bind and respond to HCO_3^- stimulation.

Nonetheless, the pH sensitivity of sea urchin sAC remained a topic of debate. Although the early studies reported strong pH sensitivity of cAMP synthesis, some of these same authors later proposed that HCO_3^- regulates sea urchin sAC instead (30, 33). Most publications do not address these conflicting results or comment on which regulatory mechanism might be more relevant, except for Tresguerres et al. (2014) (34) who discusses this issue (see below). Our Discussion section considers several possible explanations for these discrepancies. One additional important factor could be the choice of pH buffer. Some authors recommend Tris as a pH buffer (35) and used it in their experiments (36). However, Tris possesses one of the highest temperature coefficients for its pK_a value (- 0.31 $\Delta pK_a/10\ ^oC$), meaning its buffering capacity and solution pH changes with temperature (37). Although the authors adjusted the pH of the Tris buffer according to the HCO_3^- concentration, the temperature sensitivity of Tris may not have been taken into account, especially considering the reaction conditions of 30 minutes at 30 ^oC.

The first paper that coined sAC as an acid-base sensor is probably Tresguerres et al. (2010). This designation was frequently used for studies in other species (see (38) and for reviews (34, 39)). Tresguerres et al. (2014) noted that the K_m value of sea urchin sAC activation by HCO_3^- is high (if similar to that of mammalian sAC) compared with the low HCO_3^- concentrations in aquatic animals. The authors raised doubts about the physiological significance of sAC in sea urchin; alternatively, they suggested that the high K_m value and the steep pH dependence “may reflect artifacts of working with semi-purified sAC preparations or of not utilizing physiologically relevant assay conditions”.

pH or HCO_3^- sensitivity/dependence vs. pH or HCO_3^- regulation. Distinguishing between a pH-dependent reaction and a pH-regulated enzyme is essential. Enzymes may react to pH for two different reasons. First, some biochemical reactions are inherently pH-dependent or pH-sensitive because they involve the consumption or release of protons. As a result, these reactions

are inherently pH-sensitive by first physico-chemical principles. For example, the synthesis of cyclic nucleotides - such as cAMP and cGMP - by adenylyl cyclases or guanylyl cyclases, respectively, and their subsequent breakdown by phosphodiesterases both generate protons (40–43). At steady-state, when observed in an intact cell, the combined cycle of synthesis and hydrolysis produces two protons per cycle. When either sAC or PDE is activated, a rapid turnover cycle ensues, resulting in increased H^+ production. This acidification can eventually halt cAMP or cGMP production. This may explain why NHEs are often co-expressed with adenylyl cyclases (44).

Second, in proteins, the side chains of multiple amino acids can be protonated or deprotonated depending on pH and their pK_a values. These changes in protonation can slightly alter enzyme activity, but this does not necessarily mean the enzyme is *regulated* by pH. Only when the enhancement of activity is substantial can the enzyme be considered pH-regulated, and such pH sensitivity may serve a specific physiological function. Similarly, HCO_3^- or CO_3^{2-} may change water structure at the protein-water interface and, thereby, alter enzymatic activity slightly.

Given this perspective, it is worth asking how physiologically relevant a modest pH sensitivity - such as a 1.3-fold change in activity for mammalian sAC - or a similarly small HCO_3^- sensitivity in sea urchin sAC, truly is. Furthermore, even if there is a weak HCO_3^- sensitivity, it remains unclear how HCO_3^- concentrations are elevated during spawning or chemotactic navigation in sea urchin sperm. By contrast, alkalization of sea urchin sperm by chemoattractants and alkalization during spawning is well documented (27, 45) and also shown in this manuscript for *A. punctulata* sperm.

Supplementary Table 2: Overview of polymorphism in the HCO_3^--binding site in species of 13 phyla. (Excel sheet)

Overview of the polymorphism (PM) in sAC across the animal kingdom, ordered by phylum and sub-divided by class, order, or other taxonomic rank (collectively called 'group'). We focused on a few typical groups of species. *Homo sapiens* is given as reference. Species marked in green, share the same PM as *Homo sapiens*. Species or groups marked in red lack sAC (e.g., *Drosophila melanogaster*, Neoteleostei). № of genomes refers to the number of genomes available at the time of investigation.

phylum	group		№ of genomes	common name	exemplary species	polymorphism	
Chordata	Mammalia		-	Human	*Homo sapiens*	K95	R176
	Reptilia		74	Tortoises/turtles	*Chelonia mydas*	N	R
					Pelodiscus sinensis	T	R
					Terrapene carolina mexicana	N	R
				Gekko	*Gekko japonicus*	N	R
				Alligator	*Alligator mississippiensis*	N	R
				Crocodile	*Crocodylus porosus*	N	R
	Aves		532	Birds	mostly negative	-	-
				Wild Duck	*Anas platyrhynchos*	K	M
				Crested Ibis	*Nipponia nippon*	K	R
				Turkey	*Meleagris gallopavo*	K	I
				Chicken	*Gallus gallus*	K	V
	Amphibia		29	Amphibians	mostly negative	-	-
				Gaboon cecilian	*Geotrypetes seraphini*	N	R
	Fish	Other ray-finned fish	188	Zebrafish	*Danio rerio*	-	-
				Salmon	*Salmo salar*	N	R
				Herring	*Clupea harengus*	N	R
				Pike	*Esox lucius*	N	R
				Trout	*Oncorhynchus mykiss*	N	R
				Piranha	*Pygocentrus nattereri*	N	R
		Neoteleostei	523	Modern ray-finned fish	negative	-	-
		Dipnomorpha	2	Lungfish	*Protopterus annectens*	K	R
		Actinistia	1	Coelacanth	*Latimeria chalumnae*	K	R
		Chondrichthyes	11	Cartilaginous fish	*Squalus acanthias*	N	R
					Rhincodon typus	N	R
					Callorhinchus milii	N	R
	Urochordata		16	Sea squirt	*Ciona intestinalis*	K	N
	Cephalochordata		6	Lancelet	*Branchiostoma floridae*	K	N
Hemichordata	Hemichordata		2	Acorn worm	*Saccoglossus kowalevskii*	K	R
Echinodermata	Echinodermata		24	Sea urchin	*Strongylocentrotus purpuratus*	K	N
				Sea urchin	*Arbacia punctulata*	K	N
				Starfish	*Acanthaster planci*	K	N
Arthropoda	Insects	Diptera	280	Mosquito	*Aedes albopictus*	K	K
				Fly	*Drosophila melanogaster*	-	-
		Hymenoptera	225	Wasp	*Vespula vulgaris*	K	K
		Coleoptera	86	Beetle	*Tribolium castanea*	K	K
		Lepidoptera	759	Butterfly	*Papilio machaon*	K	K
		Trichoptera	8	Caddisfly	*Limnephilus lunatus*	-	-
	Crustacea		62	Water fleas	negative	-	-
			2	Barnacle	*Pollicipes pollicipes*	Q	N
	Diplura		2	Two-pronged bristletails	negative	-	-
	Collembola		33	Spring tail	*Folsomia candida*	K	S
Priapulida	Priapulida		1	Marine worm	*Priapulus caudatus*	N	R
Nematoda	Nematodes		145	Roundworms	negative	-	-
Annelida	Annelida		16	Earth worms	*Eisenia fetida*	K	N
					Owenia fusiformis	K	N
					Aporrectodea caliginosa	K	N
					Dimorphilus gyrociliatus	K	T
Mollusca	Molluscs		79	Oyster	*Crassostrea gigas*	K	N
				Octopus	*Octopus vulgaris*	-	-
				Scallop	*Patinopecten yessoensis*	K	N
Brachiopoda	Brachiopoda		1	Lamp shell	*Lingula anatina*	K	N
Cnidaria	Cnidaria		70	Corals	*Acropora danai*	K	N
					Pocillopora damicornis	K	N
				Sea anemones	*Nematostella vectensis*	K	N
					Exaiptasia pallida	K	N
Placozoa	Placozoa		1	Marine multi-cellular organism	*Trichoplax adhaerens*	K	R
Ctenophora	Ctenophora		5	Comb jellies	*Mnemiopsis leidyi*	S	N
					Pleurobrachia bachei	S	N
Porifera	Porifera		4	Sponge	*Amphimedon queenslandica*	K	T

Supplementary Table 3: Conservation of K95/R176 residues in 100 mammalian species.

Overview of mammalian sAC sequences at the sites of K95 and R176 polymorphism. For each species, the sequence of 10 amino-acid residues before and after the site of polymorphism (PM) is shown; the sequences for both polymorphisms have been concatenated. The full-length protein sequences were aligned to obtain the polymorphism sites. Hyphens represent gaps in the alignment (in *Erinaceus europaeus*, *Pteropus vampyrus*, *Vicugna Pacos*), indicating an absence of the region in the species' sequence. The sequences of 100 species were complete, whereas the sequences from 3 species showed defects and omissions in the two PM sites. Therefore, these sequences were excluded from the analysis.

Species	Sequence concatenate from alignment	PM 1	PM 2
Acinonyx jubatus (Cheetah)	KVLIFGGDILKFAGDALLALWGQAVDDVRLAQNMAQMNDVILSPN	K	R
Ailuropoda melanoleuca (Giant pand	KVLIFGGDILKFAGDALLALWGQVVDDVRLAQNMAQMNDVILSPN	K	R
Aotus nancymaae (Ma's night monke	KVLIFGGDILKFAGDALLALWGQPVDDVRLAQSMAQINDVILSPN	K	R
Balaenoptera acutorostrata scammo	KVLIFGGDILKFAGDALLALWGQAVDDVRLAQNMAQMNDVILSPN	K	R
Balaenoptera musculus (Blue whale)	KVLIFGGDILKFAGDALLALWGQAVDDVRLAQNMAQMNDVILSPN	K	R
Bison bison bison (North American p	KVLIFGGDILKFAGDALLALWGQAVDDVRLAQNMAQVNDVILSPN	K	R
Bos indicus (Zebu)	KVLIFGGDILKFAGDALLALWGQAVDDVRLAQNMAQVNDVILSPN	K	R
Bos indicus x Bos taurus (Hybrid catt	KVLIFGGDILKFAGDALLALWGQAVDDVRLAQNMAQVNDVILSPN	K	R
Bos mutus grunniens (Wild yak) (Bos	KVLIFGGDILKFAGDALLALWGQAVDDVRLAQNMAQVNDVILSPN	K	R
Bos taurus (Bovine)	KVLIFGGDILKFAGDALLALWGQAVDDVRLAQNMAQVNDVILSPN	K	R
Callithrix jacchus (White-tufted-ear m	KVLIFGGDILKFAGDALLALWGPPVDDVRLAQSMAQTNDVILSPN	K	R
Callorhinus ursinus (Northern fur seal	KVLIFGGDILKFAGDALLALWGQAVDDVRLAQNMAQMNDVILSPN	K	R
Camelus bactrianus (Bactrian camel)	KVLIFGGDILKFAGDALLALWGQAVDDVRLAQNMAQMNDVILSPN	K	R
Camelus ferus (Wild bactrian camel)	KVLIFGGDILKFAGDALLALWGQAVDDVRLAQNMAQMNDVILSPN	K	R
Canis lupus dingo (dingo)	KVLLFGGDILKFAGDALLALWGQAVDDVRLAQNMAQMNDVILSPN	K	R
Canis lupus familiaris (Dog) (Canis fa	KVLLFGGDILKFAGDALLALWGQAVDDVRLAQNMAQMNDVILSPN	K	R
Capra hircus (Goat)	KVLIFGGDILKFAGDALLALWGQAVDDVRLAQNMAQVNDVILSPN	K	R
Carlito syrichta (Philippine tarsier) (T	KVLIFGGDILKFAGDALLALWGQTVDDVRHAQGMAQMNDVILSQN	K	R
Castor canadensis (American beave	KVLIFGGDILKFAGDALLALWGQAVNDVRLAQNMAQMNDVILSPN	K	R
Cavia porcellus (Guinea pig)	KVLLFGGDILKFAGDALLALWGQAVDDVRLAQNMAQMNDVILSPT	K	R
Cebus imitator (Panamanian white-fa	KVLIFGGDILKFAGDALLALWGQPVDDVRLAQNMAQINDVILSPN	K	R
Cercocebus atys (Sooty mangabey)	KVLIFGGDILKFAGDALLALWGQAVDDVRLAQNMAQMNDVILSPN	K	R
Chinchilla lanigera (Long-tailed chinc	KVLLFGGDILKFAGDALLALWGQAVEDVRMAQNMALMNDVILSPN	K	R
Chlorocebus sabaeus (Green monke	KVLIFGGDILKFAGDALLALWGQAVDDVRLAQNMAQMNDVILSPN	K	R
Chrysochloris asiatica (Cape golden	KVLIFGGDILKFAGDALLALWGQPVDDVRFAQNMAQMNEVILSPN	K	R
Cricetulus griseus (Chinese hamster)	KVLIFGGDILKFAGDALLALWGQAVDDVRLAQNMAHMNDVILSPN	K	R
Crocuta crocuta (Spotted hyena)	KVLIFGGDILKFAGDALLALWGAEIESDAQPTEPPRCPETVLILI	K	A
Delphinapterus leucas (Beluga whale	KVLIFGGDILKFAGDALLALWGQAVDDVRLAQNMAQMNDVILSPN	K	R
Dipodomys ordii (Ord's kangaroo rat	KVLIFGGDILKFAGDALLALWGQAVDDVRLAQGMAQMNDVILSPN	K	R
Equus asinus (Donkey) (Equus africa	KVLIFGGDIIKFAGDALLALWGQAVDDVRLAQNMAQMNDVILSPN	K	R
Equus caballus (Horse)	KVLIFGGDIIKFAGDALLALWGQAVDDVRLAQNMAQMNDVILSPN	K	R
Erinaceus europaeus (Western Euro	---	-	-
Felis catus (Cat) (Felis silvestris catu	KVLIFGGDILKFAGDALLALWGQAVDDVRLAQNLAQMNDVILSPN	K	R
Gorilla gorilla gorilla (Western lowlan	KVLIFGGDILKFAGDALLALWGQAVDDVRLAQNMAQMNDVILSPN	K	R
Heterocephalus glaber (Naked mole	KVLLFGGDILKFAGDALLALWGQAVDDVRLAQNLAQMNDVILSPN	K	R
Hipposideros armiger (Great Himala	KVLLFGGDILKFAGDALLALWGQAVEDVRLAQNMAQMDDVILSPN	K	R
Homo sapiens (Human)	KVLIFGGDILKFAGDALLALWGQAVDDVRLAQNMAQMNDVILSPN	K	R
Ictidomys tridecemlineatus (Thirteen-	KVLMFGGDILKFAGDALLALWGQAVDDVRLAQNMARMNDVILSPN	K	R
Jaculus jaculus (Lesser Egyptian jerb	KVLVFGGDILKFAGDALLALWGQAVDDVRLAQNMAQMNDVILSPN	K	R
Leptonychotes weddellii (Weddell se	KVLIFGGDILKFAGDALLALWGQAVDDVRLAQNMAQMNDVILSPN	K	R
Lipotes vexillifer (Yangtze river dolphi	KVLIFGGDILKFAGDALLALWGQAVDDVRLAQNMAQMNDVILSPN	K	R
Loxodonta africana (African elephant	KVLIFGGDILKFAGDALLALWGQAVDDVRFAQNMAQMNDVILSPN	K	R
Lynx canadensis (Canada lynx) (Felis	KVLIFGGDILKFAGDALLALWGQAVDDVRLAQNMAQMNDVILSPN	K	R
Macaca fascicularis (Crab-eating ma	KVLIFGGDILKFAGDALLALWGQAVDDVRLAQNMAQMNDVILSPN	K	R
Macaca mulatta (Rhesus macaque)	KVLIFGGDILKFAGDALLALWGQAVDDVRLAQNMAQMNDVILSPN	K	R
Macaca nemestrina (Pig-tailed maca	KVLIFGGDILKFAGDALLALWGQAVDDVRLAQNMAQMNDVILSPN	K	R
Mandrillus leucophaeus (Drill) (Papio	KVLIFGGDILKFAGDALLALWGQAVDDVRLAQNMAQMNDVILSPN	K	R
Marmota marmota marmota (Alpine	KVLMFGGDILKFAGDALLALWGQAVDDVRLAQNMARMNDVILSPN	K	R
Mesocricetus auratus (Golden hamst	KVLIFGGDILKFAGDALLALWGQAVDDVRLAQNMAHMNDVILSPN	K	R
Microcebus murinus (Gray mouse ler	KVLIYGGDILKFAGDALLALWGQTVDDVRRAQNMAQMNDVILSPN	K	R
Monodelphis domestica (Gray short-t	KVLIYGGDILKFAGDALLALWGQAVEDVRFAQNMAQMNEIILSPN	K	R
Monodon monoceros (Narwhal) (Cer	KVLIFGGDILKFAGDALLALWGQAVDDVRLAQNMAQMNDVILSPN	K	R
Moschus moschiferus (Siberian musl	KVLIFGGDILKFAGDALLALWGQAVDDVRLAQNMAQVNDVILSPN	K	R
Mus caroli (Ryukyu mouse) (Ricefield	KVLIFGGDILKFAGDALLALWGQAVDDVRLAQNMAQMNDVILSPN	K	R
Mus musculus (Mouse)	KVLIFGGDILKFAGDALLALWGQAVDDVRLAQNMAQMNDVILSPN	K	R
Mustela putorius furo (European dom	KVLIFGGDILKFAGDALLALWGQAVEDVRLAQNMAQMNDVILSPN	K	R
Myotis lucifugus (Little brown bat)	KVLIFGGDILKFAGDALLALWGQTVDDVRLAQNMAQMNDVILSPN	K	R

Species	Sequence concatenate from alignment	PM 1	PM 2
Nannospalax galili (Northern Israeli b	KVLIFGGDILKFAGDALLALWGQTVDDVRVAQNMAQMNDVILSPE	K	R
Neophocaena asiaeorientalis asiaec	KVLIFGGDILKFAGDALLALWGQAVDDVRLAQNMAQMNDVILSPN	K	R
Neomonachus schauinslandi (Hawaii	KVLIFGGDILKFAGDALLALWGQAVDDVRLAQNMAQMNDVILSPN	K	R
Neovison vison (American mink) (Mu	KVLIFGGDILKFAGDALLALWGQAVEDVRLAQNMAQMNDVILSPN	K	R
Nomascus leucogenys (Northern whit	KVLIFGGDILKFAGDALLALWGQAVDDVRLAQNMAQMNDVILSPN	K	R
Octodon degus (Degu) (Sciurus degu	KVLLFGGDILKFAGDALLALWGQAVEDVRNAQNMAQMNDVILSPN	K	R
Odobenus rosmarus divergens (Paci	KVLIFGGDILKFAGDALLALWGQAVDDVRLAQNMAQMNDVILSPN	K	R
Odocoileus virginianus texanus	KVLIFGGDILKFAGDALLALWGQAVDDVRLAQNMAQVNDVILSPN	K	R
Ornithorhynchus anatinus (Duckbill pl	KVLIYGGDILKFAGDALLALWGQAVEDVRFAQGLAQMNEVILSPN	K	R
Orycteropus afer afer	KVLIFGGDILKFAGDALLALWGQTVDDVRFAQNMAQMNEVILSPN	K	R
Oryctolagus cuniculus (Rabbit)	KVLIFGGDILKFAGDALLALWGQAVDDVRLAQNMARMNDVILSPN	K	R
Otolemur garnettii (Small-eared gala	KVLIYGGDILKFAGDALLALWGQVVDDVRLAQGMAQMNDVILSPN	K	R
Ovis aries (Sheep)	KVLIFGGDILKFAGDALLALWGQAVDDVRLAQNMAQVNDVILSPN	K	R
Panthera leo (Lion)	KVLIFGGDILKFAGDALLALWGQAVDDVRLAQNMAQMNDVILSPN	K	R
Pan paniscus (Pygmy chimpanzee) (	KVLIFGGDILKFAGDALLALWGQAVDDVRLAQNMAQMNDVILSPN	K	R
Panthera pardus (Leopard) (Felis pa	KVLIFGGDILKFAGDALLALWGQAVDDVRLAQNMAQMNDVILSPN	K	R
Panthera tigris altaica (Siberian tiger	KVLIFGGDILKFAGDALLALWGQAVDDVRLAQNMAQMNDVILSPN	K	R
Pan troglodytes (Chimpanzee)	KVLIFGGDILKFAGDALLALWGQAVDDVRLAQNMAQMNDVILSPN	K	R
Papio anubis (Olive baboon)	KVLIFGGDILKFAGDALLALWGQAVDDVRLAQNMAQMNDVILSPN	K	R
Peromyscus maniculatus bairdii (Pra	KVLIFGGDILKFAGDALLALWGQAVDDVRLAQNMAQMNDVILSPN	K	R
Phascolarctos cinereus (Koala)	KVLIFGGDILKFAGDALLALWGQAVEDVRFAESMAQMNDTILSPN	K	R
Phodopus roborovskii (Roborovski's	KVLIFGGDILKFAGDALLALWGQAVDDVRLAQNMAHMNDVILSPN	K	R
Phocoena sinus (Vaquita)	KVLIFGGDILKFAGDALLALWGQAVDDVRLAQNMAQMNDVILSPN	K	R
Phyllostomus discolor (pale spear-nc	KVLLFGGDILKFAGDALLALWGQAVDDVRLAQNMAQMNDVILSPN	K	R
Physeter macrocephalus (Sperm wha	KVLIFGGDILKFAGDALLALWGQAVDDVRLAQNMAQMNDVILSPN	K	R
Pongo abelii (Sumatran orangutan) (	KVLIFGGDILKFAGDALLALWGQAVDDVRLAQNMAQMNDVILSPN	K	R
Propithecus coquereli (Coquerel's sit	KVLIYGGDILKFAGDALLAMWGQTVDDVRRAQNMAQMNDVILSPN	K	R
Pteropus vampyrus (Large flying fox)	---------------------GQAVEDVRLAQNMAQMNDVILSPN	-	R
Puma concolor (Mountain lion) (Felis	KVLIFGGDILKFAGDALLALWGQAIGWRRALPFMAQMSDVILSTK	K	R
Rattus norvegicus (Rat)	KVLIFGGDILKFAGDALLALWGQAVDDVRLAQNMAQMNDVILSPN	K	R
Rhinopithecus bieti (Black snub-nose	KVLIFGGDILKFAGDALLALWGQAVDDVRLAQNMAQMNDVILSPN	K	R
Rhinolophus ferrumequinum (Greater	KVLIFGGDILKFAGDSLLALWGQVVDDVRLAQNLAQMNDVILSPN	K	R
Rhinopithecus roxellana (Golden snul	KVLIFGGDILKFAGDALLALWGQAVDDVRLAQNMAQMNDVILSPN	K	R
Saimiri boliviensis boliviensis (Bolivia	KVLIFGGDILKFAGDALLALWGQPVDDVRLAQSMAQINDVILSPN	K	R
Sapajus apella (Brown-capped capu	KVLIFGGDILKFAGDALLALWGQPVDDVRLAQNMAQINDVILSPN	K	R
Sarcophilus harrisii (Tasmanian devi	KVLIFGGDILKFAGDALLALWGQAVEDVRFAQSMAQMNDIILSPN	K	R
Sciurus vulgaris (Eurasian red squirre	KVLMFGGDILKFAGDALLALWGQAVDDVRLAQNMARMNDVILSPN	K	R
Sus scrofa (Pig)	KVLIFGGDILKFAGDALLALWGQAVDDVRRAQNMAQMNDVILSPN	K	R
Theropithecus gelada (Gelada baboo	KVLIFGGDILKFAGDALLALWGQAVDDVRLAQNMAQMNDVILSPN	K	R
Trichechus manatus latirostris (Florid	KVLLFGGDILKFAGDALLALWGQAVDDVRFAQNMAQMNDVILSPN	K	R
Tursiops truncatus (Atlantic bottle-nos	KVLIFGGDILKFAGDALLALWGQAVDDVRLAQNMAQMNDVILSPN	K	R
Ursus maritimus (Polar bear) (Thalarc	KVLIFGGDILKFAGDALLALWGQAVDDVRIAQNMAQMNDVILSPN	K	R
Vicugna pacos (Alpaca) (Lama pacc	---------------------GQAVHDVRLAQNMAQMNDVILSPN	-	R
Vombatus ursinus (Common womba	KVLIFGGDILKFAGDALLALWGQAVEDVRFAQSMAQMNDTILSPN	K	R
Vulpes vulpes (Red fox)	KVLLFGGDILKFAGDALLALWGQTVDDVRLAQNMAQMNDVILSPN	K	R
Zalophus californianus (California sea	KVLIFGGDILKFAGDALLALWGQAVDDVRLAQNMAQMNDVILSPN	K	R

Supplementary Table 4: Variation of K/R polymorphism in fish. (Excel sheet)

Overview of fish species possessing sAC. For each species, the respective sequence of 10 amino-acid residues before and after the site of polymorphism (PM) is shown; PM sites are marked in red. *Homo sapiens* (highlighted in grey) is given as reference. Species that had previously been annotated for sAC proteins are bolded. For species where multiple isoforms of sAC were found (e.g., *Ictarus punctatus*), all differing PMs are listed. The full-length protein sequences were aligned to obtain the PM sites; hyphens represent gaps in the alignment.

species	group	PM1 region	PM1	PM2 region	PM2
Homo sapiens	**mammals**	**KVLIFGGDILKFAGDALLALW**	**K**	**LVIGQAVDDVRLAQNMAQMND**	**R**
Latimeria chalumnae	**lobe-finned fish**	**HYLTQGKKVFKHSSDALLALW**	**K**	**VVIGRAVDEVRLAEGLALANT**	**R**
Protopterus annectens	**lobe-finned fish**	**HILIAGGDILKFAGDALLALW**	**K**	**AVLGLAIDGIRKAEGLATAGD**	**R**
Amblyraja radiata	**Chondrichthyes**	**HILRAGGDIVNYAGDAMLALW**	**N**	**ALIGRAIDEVRKAEGLASANT**	**R**
Callorhinchus milii	Chondrichthyes	HILQAGGDIVNYAGDAMLALW	N	ALIGCAVDEVRKAEGLALANT	R
Carcharodon carcharias	Chondrichthyes	HILQAGGDIVNYAGDAMLALW	N	ALIGRAVDEVRKAEGLASANT	R
Chiloscyllium plagiosum	Chondrichthyes	HILRAGGDVVNYAGDAMLALW	N	ALIGRAVDEVRKAEGLASANT	R
Chiloscyllium punctatum	Chondrichthyes	HILRAGGDVVNYAGDAMLALW	N	ALIGRAVDEVRKAEGLASANT	R
Hemiscyllium ocellatum	Chondrichthyes	HILRAGGDVVNYAGDAMLALW	N	ALIGRAVDEVRKAEGLASANT	R
Hydrolagus affinis	Chondrichthyes	HILRAGGDIVNYAGDAMLALW	N	ALIGRAVDEVRKAEGLALAHT	R
Rhincodon typus	**Chondrichthyes**	**HILRAGGDIVNYAGDAMLALW**	**N**	**ALIGRAVDEVRKAEGLASANT**	**R**
Scyliorhinus canicula	Chondrichthyes	DCLTLCMIIFFPTGDAMLALW	F	ALIGRAVDEVRKAEGLASANT	R
Scyliorhinus torazame	Chondrichthyes	HILRAGGDIVNYAG-AMLALW	N	ALIGRAVDEVRKAEGLASANT	R
Squalus acanthias	**Chondrichthyes**	**HILRAGGDIVNYAGDAMLALW**	**N**	**ALIGRAVDEVRKAEGLASANT**	**R**
Acipenser ruthenus	early ray-finned fish: Chondrostei	YILTSGGDILNYAGDAILALW	N	VVIGRAVDEVRLAEGLAVAHS	R
Polyodon spathula	early ray-finned fish: Chondrostei	YILTSGGDILNYAGDAILALW	N	VVIGHAVDEVRLAEGLAVAHS	R
Lepisosteus oculatus	**early ray-finned fish: Holostei**	**HILAAGGDILNYAGDAILALW**	**N**	**VVIGRAVDEVRLAEGLAVAST**	**R**
Ageneiosus marmoratus	Otomorpha: Characiphysae	-MRAYGNHMVLSAGDAILAMW	L	LVIGPAVDELLSAEVLAEHAD	L
Ageneiosus marmoratus	Otomorpha: Characiphysae	-MWMWMCYMILSADGSILALW	L	VVLGPAVEDLHSAVVLAEQAD	H
Ameiurus melas	Otomorpha: Characiphysae	-MTVGKCYIILPAGDAILAEW	L	AVIGPAVEEIRSAQALLEHGV	R
Ameiurus melas	Otomorpha: Characiphysae	CILEEGGDILNYAGDAILAQW	N	AVIGPAVDEVRLTEPLANPGD	R
Clarias batrachus	Otomorpha: Characiphysae	FLTQASTWIFFISGDSILAQW	F	VITGPALNEVRLAEALANPGD	R
Clarias macrocephalus	Otomorpha: Characiphysae	EKVLAQGSIWIFSGDSILAQW	I	VITGPAVNEVRLAEPLANAGD	R
Clarias magur	**Otomorpha: Characiphysae**	**CILEEGGDILNYAGDSILAQW**	**N**	**VITGPALNEVRLAEPLANPGD**	**R**
Clarias magur	Otomorpha: Characiphysae	FLTQASTWIFFISGDSILAQW	F	VITGPALNEVRLAEPLANPGD	R
Colossoma macropomum	Otomorpha: Characiphysae	QILADGGDILNYAGDAILALW	N	VVIGRAVDEVRLAESLAVAGT	R
Hepsetus odoe	Otomorpha: Characiphysae	HILLDGGDILNYAGDAILALW	N	VVIGHAVDEVRLAEGLAEAST	R
Ictalurus punctatus	**Otomorpha: Characiphysae**	**CILEEGGDILNYAGDAILAQW**	**N**	**AVIGPAVDEVRLTEPLANPGD**	**R**
Ictalurus punctatus	Otomorpha: Characiphysae	CKLLHRFDKIFPPGDAILAQW	F	AVIGPAVDEVRLTEPLANPGD	R
Ictalurus punctatus	Otomorpha: Characiphysae	RTSVHQRYVILLAGDAILAEW	L	AVIGPAVEEIRSIQARMQNGD	R
Ompok bimaculatus	Otomorpha: Characiphysae	CILEEGGDILNFPGDAILAQW	N	VITGPAVDEVRMTESLANPGD	R
Pangasianodon hypophthalmus	Otomorpha: Characiphysae	DYMLNGCRMILPADDVILAQW	L	VVFGPAVEDIRSAEALVEPGK	R
Pangasianodon hypophthalmus	Otomorpha: Characiphysae	CILEEGGDILNYAGDAILAQW	N	VIIGPAVEEVRLAEPLANPGD	R
Pangasianodon hypophthalmus	Otomorpha: Characiphysae	RMTVHGCYMIVPAGDAILAEW	V	VVIGPAVDEMRSAEVLAKHGN	R
Pygocentrus nattereri	Otomorpha: Characiphysae	RKTVHGCCIILSAGDAILALW	L	IVIGRAVDEVRLAESLAVAGT	R
Silurus glanis	Otomorpha: Characiphysae	CKL---------PGDAILAQW	-	VITGPAVDEVRMNESLANPGD	R
Silurus meridionalis	Otomorpha: Characiphysae	------------PGDAILAQW	-	VITGPAVDEVRMNESLANPGD	R
Alosa alosa	Otomorpha: Clupeiformes	RKLYTSHLIFVFTGDAILALW	V	VVIGRAVDEVRLAEGLAVAGN	R
Alosa sapidissima	Otomorpha: Clupeiformes	RKLYTSHLIFVFTGDAILALW	V	VVIGRAVDEVRLAEGLAVAGN	R
Clupea harengus	Otomorpha: Clupeiformes	YILAAGGDILNYAGDAILALW	N	VVIGRAVDEVRLAEGLAVAGN	R
Coilia nasus	Otomorpha: Clupeiformes	HILAAGGDILNYAGDAILALW	N	VVIGRAVDEVRLAEGLAVAGN	R
Sardina pilchardus	Otomorpha: Clupeiformes	HILAAGGDILNYAGDAILSLW	N	VVIGRAVDEVRLAEGLAVAGN	R
Tenualosa ilisha	Otomorpha: Clupeiformes	HLISFS------TGDAILALW	-	VVIGRAVDEVRLAEGLAVAGN	R
Tenualosa ilisha	Otomorpha: Clupeiformes	HILAAGGDILNYAGDAILALW	N	VVIGRAVDEVRLAEGLAVAGN	R
Chanos chanos	**Otomorpha: Gonorynchiformes**	**YILAAGGDVLNYAGDAILALW**	**N**	**VVIGRAVDEVRLAEGLAEAST**	**R**
Chanos chanos	Otomorpha: Gonorynchiformes	YYLMCVWHDFSTTGDAILALW	S	VVIGRAVDEVRLAEGLAEAST	R
Chanos chanos	Otomorpha: Gonorynchiformes	QSIFYKKSILVYS-GVTSALW	V	RRYDAAMLTQIALASLAKVNI	I
Aplochiton taeniatus	Protacanthopterygii	HILASGGDILNYAGDAILALW	N	VVIGRAVDEVRLAEGLAVAGS	R
Coregonus clupeaformis	Protacanthopterygii	HILDAGGDILNYAGDAILALW	N	VVIGRAVDEVRLAEG-AVAST	R
Dallia pectoralis	Protacanthopterygii	HILAAGGDILNYAGDAILALW	N	VVIGRAVDEVRLAEGLAAAST	R
Esox lucius	Protacanthopterygii	YILASGGDILNFAGDAILALW	N	LVIGRAVDEVRLAEGLAVAGT	R
Esox masquinongy	Protacanthopterygii	YILASGGDILNFAGDAILALW	N	LVIGRAVDEVRLAEGLAAAST	R
Esox niger	Protacanthopterygii	YILASGGDILNFAGDAILALW	N	LVIGRAVDEVRLAEGLAEAST	R
Hucho hucho	Protacanthopterygii	RFLLVMAKQLLITGDAILALW	L	VVIGRAVDEVRLAEGLAVAST	R
Novumbra hubbsi	Protacanthopterygii	HILASGGDVLNYAGDAILALW	N	LVIGRAVDEVRLAEGLATPST	R
Oncorhynchus gorbuscha	Protacanthopterygii	TILLVMAKPLLITGDAILALW	L	VVIGRAVDEVRLAEGLAVVST	R
Oncorhynchus gorbuscha	Protacanthopterygii	HILDAGGDILNYAGDAILALW	N	VVIGRAVDEVRLAEGLAVAST	R
Oncorhynchus keta	Protacanthopterygii	RKLAMVLLLLLKQSDAILALW	L	VVIGRAVDEVRLAEGLAVAST	R
Oncorhynchus keta	Protacanthopterygii	HILDAGGDILNYAGDAILALW	N	VVIGRAVDEVRLAEGLAVAST	R
Oncorhynchus kisutch	**Protacanthopterygii**	**HILDAGGDILNYAGDAILALW**	**N**	**VVIGRAVDEVRLAEGLAVAST**	**R**
Oncorhynchus kisutch	Protacanthopterygii	RQLFMAKQLL-ITGDAILALW	-	VVIGRAVDEVRLAEGLAVAST	R
Oncorhynchus mykiss	Protacanthopterygii	RQLFMAKQLL-ITGDAILALW	-	VVIGRAVDEVRLAEGLAVAST	R
Oncorhynchus nerka	**Protacanthopterygii**	**HILDAGGDILNYAGDAILALW**	**N**	**VVIGRAVDEVRLAEGLAVAST**	**R**
Oncorhynchus nerka	Protacanthopterygii	RQLFMAKQLL-ITGDAILALW	-	VVIGRAVDEVRLAEGLAVAST	R
Oncorhynchus tshawytscha	Protacanthopterygii	RQLFMAKQLL-ITGDAILALW	-	VVIGRAVDEVRLAEGLAVAST	R
Oncorhynchus tshawytscha	Protacanthopterygii	RKL-----AVVSLSDAILALW	V	VVIGRAVDEVRLAEGLAVAST	R
Salmo salar	**Protacanthopterygii**	**HILDAGGDILNYAGDAILALW**	**N**	**VVIGRAVDEVRLAEGLAVAST**	**R**
Salmo salar	Protacanthopterygii	RFLLFMAKQLLITGDAILALW	L	VVIGRAVDEVRLAEGLAVAST	R
Salmo trutta	**Protacanthopterygii**	**HILDAGGDILNYAGDAILALW**	**N**	**VVIGRAVDEVRLAEGLAVAST**	**R**
Salvelinus alpinus	**Protacanthopterygii**	**HILDAGGDILNYAGDAILALW**	**N**	**VVIGRAVDEVRLAEGLAVAST**	**R**
Salvelinus alpinus	Protacanthopterygii	RFLLFMAKQLLITGDAILALW	L	VVIGRAVDEVRLAEGLAVAST	R
Salvelinus namaycush	Protacanthopterygii	RFLLFMAKQLLITGDAILALW	L	VVIGRAVDEVRLAEGLAVAST	R
Thymallus thymallus	Protacanthopterygii	RKLVVSTKQLFITGDAILALW	F	VVIGRAVDEVRLAEGLAVAST	R
Borostomias antarcticus	Stomiatii	HILASGGDVLNYAGDAVLALW	N	VVIGRAVDEVRLAEGLALAST	R
Hypomesus nipponensis	Stomiatii	HILDAGGDILNYA--------	N	VVIGRAVDEVRLAEGLAVASS	R
Mallotus villosus	Stomiatii	HILDAGGDILNYAGDAILALW	N	VVIGRAVDEVRLAEGLAVASS	R
Osmerus eperlanus	Stomiatii	HILDAGGDILNYAGDAILALW	N	VVIGRAVDEVRLAEGLAVASS	R
Plecoglossus altivelis	Stomiatii	HILDAGGDILNYAGDAILALW	N	VVIGRAVDEVRLAEGLAVAGS	R
Thaleichthys pacificus	Stomiatii	HILDAGGDILNYAGDAILALW	N	VVIGRAVDEVRLAEGLAVASS	R

Supplementary Table 5: Composition of seminal fluid of *A. punctulata* and *S. salar*

	A. punctulata[1]		***S. salar***[1]	
component	seminal fluid[2]	artificial seawater[3]	seminal fluid[4]	fresh water[5]
pH	6.8 ± 0.25	7.8	8.3	8.1
Na^{+}	430.8 ± 12.8	423	110	0.5
K^{+}	25.7 ± 2.1	9	37	0.1
Ca^{2+}	9.5 ± 0.2	9.3	0.65	0.8
Mg^{2+}	51.1 ± 1.2	48.5	1.15	0.2
Cl^{-}	514.9 ± 10.8	496.5	109	0.7
osmolality	1058.7	1011.8	265	3-4

[1]concentrations in mM, except pH; [2]ions: n = 6 experiments, pH: n = 5 experiments; [3](Hamzeh et al., 2019); [4](Rosengrave et al., 2009, *Comp Biochem Physiol* 152, 123-129); [5]Wahnbachtalsperrenverband Siegburg, Germany.

Supplementary Table 6: Primers for *Aps*AC$_t$ mutants

Mutation, Forward (F) or Reverse (R)	Sequence
K117A F	TCCTCGCATTCGCAGGCGATGCCTTCCTGGCGC
K117A R	CCTGCGAATGCGAGGACGTCTCCCTCTGTGCCC
K117N F	GTCCTCAACTTCGCAGGCGATGCCTTCCTGG
K117N R	TGCGAAGTTGAGGACGTCTCCCTCTGTGCCC
K117R F	GTCCTCAGGTTCGCAGGCGATGCCTTCCTGG
K117R R	TGCGAACCTGAGGACGTCTCCCTCTGTGCCC
K214A F	TCACCCGCAATGTGGGCCTACTGTCCCGACC
K214A R	CCACATTGCGGGTGAGAGGATGACGTCACCTGA
K214Q F	CACCCCAGATGTGGGCCTACTGTCCCGAC
K214Q R	CCACATCTGGGGTGAGAGGATGACGTCACCT
K214R F	CACCCAGGATGTGGGCCTACTGTCCCGAC
K214R R	CCACATCCTGGGTGAGAGGATGACGTCACCT
H222A F	CCGACGCAGACCTCATCGACCATACCTTCAAG
H222A R	AGGTCTGCGTCGGGACAGTAGGCCCACATC
H222R F	CCGACAGGGACCTCATCGACCATACCTTCAAG
H222R R	AGGTCCCTGTCGGGACAGTAGGCCCACATC
H222N F	CCGACAACGACCTCATCGACCATACCTTCAAG
H222N R	AGGTCGTTGTCGGGACAGTAGGCCCACATC
N198R F	AGGCTAACATCGCCGAGAAGTTTGCCATGTCAGGTG
N198R R	TCGGCGATGTTAGCCTCCAGTACTGCTGGCCCA

References

1. C. J. Brokaw, Regulation of sperm flagellar motility by calcium and cAMP-dependent phosphorylation. *J Cell Biochem* **35**, 175–184 (1987).

2. C. J. Brokaw, Cyclic AMP-dependent Activation of Sea Urchin and Tunicate Sperm Motility. *Annals of the New York Academy of Sciences* **438**, 132–141 (1984).

3. E. Birney, M. Clamp, R. Durbin, GeneWise and Genomewise. *Genome Res.* **14**, 988–995 (2004).

4. K. Katoh, K. Misawa, K. Kuma, T. Miyata, MAFFT: a novel method for rapid multiple sequence alignment based on fast Fourier transform. *Nucleic Acids Res* **30**, 3059–3066 (2002).

5. K. Katoh, D. M. Standley, MAFFT multiple sequence alignment software version 7: improvements in performance and usability. *Mol Biol Evol* **30**, 772–780 (2013).

6. A. Sali, T. L. Blundell, Comparative protein modelling by satisfaction of spatial restraints. *J Mol Biol* **234**, 779–815 (1993).

7. C. Steegborn, T. N. Litvin, L. R. Levin, J. Buck, H. Wu, Bicarbonate activation of adenylyl cyclase via promotion of catalytic active site closure and metal recruitment. *Nat Struct Mol Biol* **12**, 32–37 (2005).

8. S. Kleinboelting, *et al.*, Crystal structures of human soluble adenylyl cyclase reveal mechanisms of catalysis and of its activation through bicarbonate. *Proc Natl Acad Sci U S A* **111**, 3727–3732 (2014).

9. T. Meyer, E.-W. Knapp, pKa values in proteins determined by electrostatics applied to molecular dynamics trajectories. *J Chem Theory Comput* **11**, 2827–2840 (2015).

10. M. Mirdita, *et al.*, ColabFold: making protein folding accessible to all. *Nat Methods* **19**, 679–682 (2022).

11. M. Mirdita, M. Steinegger, J. Söding, MMseqs2 desktop and local web server app for fast, interactive sequence searches. *Bioinformatics* **35**, 2856–2858 (2019).

12. J. M. Swails, D. M. York, A. E. Roitberg, Constant pH Replica Exchange Molecular Dynamics in Explicit Solvent Using Discrete Protonation States: Implementation, Testing, and Validation. *J. Chem. Theory Comput.* **10**, 1341–1352 (2014).

13. D. A. Case, T. E. Cheathem III, C. Simmerling, A. Roitberg, Amber 2023 Reference Manual. (2023).

14. T. Tubiana, J.-C. Carvaillo, Y. Boulard, S. Bressanelli, TTClust: A Versatile Molecular Simulation Trajectory Clustering Program with Graphical Summaries. *J. Chem. Inf. Model.* **58**, 2178–2182 (2018).

15. J. H. Zippin, *et al.*, CO2/HCO3−- and Calcium-regulated Soluble Adenylyl Cyclase as a Physiological ATP Sensor. *J Biol Chem* **288**, 33283–33291 (2013).

16. H. Liu, J. H. Naismith, An efficient one-step site-directed deletion, insertion, single and multiple-site plasmid mutagenesis protocol. *BMC Biotechnology* **8**, 91 (2008).

17. K. Kundert, Wellmap: a file format for microplate layouts. *BMC Research Notes* **14**, 164 (2021).

18. T. Braun, R. F. Dods, Development of a Mn-2+-sensitive, "soluble" adenylate cyclase in rat testis. *Proc Natl Acad Sci U S A* **72**, 1097–1101 (1975).

19. L. H. Bookbinder, G. W. Moy, V. D. Vacquier, Identification of sea urchin sperm adenylate cyclase. *J Cell Biol* **111**, 1859–1866 (1990).

20. M. Mourelle, I. Vargas, A. Darszon, Adenylate cyclase activity of membrane fractions isolated from sea urchin sperm. *Gamete Research* **9**, 87–97 (1984).

21. D. L. Garbers, Characterization of Sea Urchin Sperm Adenylate Cyclase. *Biology of Reproduction* **16**, 377–384 (1977).

22. H. Hamzeh, *et al.*, Kinetic and photonic techniques to study chemotactic signaling in sea urchin sperm. *Methods in Cell Biology* **151**, 487 (2019).

23. J. Bond, J. Varley, Use of flow cytometry and SNARF to calibrate and measure intracellular pH in NS0 cells. *Cytometry A* **64**, 43–50 (2005).

24. S. Chow, D. Hedley, I. Tannock, Flow cytometric calibration of intracellular pH measurements in viable cells using mixtures of weak acids and bases. *Cytometry* **24**, 360–367 (1996).

25. D. A. Eisner, *et al.*, A novel method for absolute calibration of intracellular pH indicators. *Pflugers Arch* **413**, 553–558 (1989).

26. P. Swietach, *et al.*, Hydrogen ion dynamics in human red blood cells. *J Physiol* **588**, 4995–5014 (2010).

27. R. Seifert, *et al.*, The Cat Sper channel controls chemosensation in sea urchin sperm. *The EMBO Journal* **34**, 379–392 (2015).

28. V. Hagen, *et al.*, Highly Efficient and Ultrafast Phototriggers for cAMP and cGMP by Using Long-Wavelength UV/Vis-Activation. *Angew Chem Int Ed Engl* **40**, 1045–1048 (2001).

29. S. P. Cook, D. F. Babcock, Activation of Ca2+ permeability by cAMP is coordinated through the pHi increase induced by speract. *Journal of Biological Chemistry* **268**, 22408–22413 (1993).

30. C. Beltrán, O. Zapata, A. Darszon, Membrane Potential Regulates Sea Urchin Sperm Adenylylcyclase. *Biochemistry* **35**, 7591–7598 (1996).

31. M. Nomura, C. Beltrán, A. Darszon, V. D. Vacquier, A soluble adenylyl cyclase from sea urchin spermatozoa. *Gene* **353**, 231–238 (2005).

32. V. D. Vacquier, A. Loza-Huerta, J. García-Rincón, A. Darszon, C. Beltrán, Soluble Adenylyl Cyclase of Sea Urchin Spermatozoa. *Biochim Biophys Acta* **1842**, 2621–2628 (2014).

33. C. Beltrán, *et al.*, Particulate and soluble adenylyl cyclases participate in the sperm acrosome reaction. *Biochem Biophys Res Commun* **358**, 1128–1135 (2007).

34. M. Tresguerres, K. L. Barott, M. E. Barron, J. N. Roa, Established and potential physiological roles of bicarbonate-sensing soluble adenylyl cyclase (sAC) in aquatic animals. *J Exp Biol* **217**, 663–672 (2014).

35. M. Tresguerres, C. Salmerón, "Chapter Seventeen - Molecular, Enzymatic, and Cellular Characterization of Soluble Adenylyl Cyclase From Aquatic Animals" in *Methods in Enzymology*,

Marine Enzymes and Specialized Metabolism - Part B., B. S. Moore, Ed. (Academic Press, 2018), pp. 525–549.

36. M. Tresguerres, *et al.*, Bicarbonate-sensing soluble adenylyl cyclase is an essential sensor for acid/base homeostasis. *Proceedings of the National Academy of Sciences* **107**, 442–447 (2010).

37. G. Capitaine, *et al.*, pHt measurements of TRIS buffer solutions in an artificial seawater matrix in the salinity range 5–40 and temperature range 5–40 °C. Part 1: Measurements and data fitting. *Marine Chemistry* **273**, 104551 (2025).

38. C. Salmerón, *et al.*, Molecular and biochemical characterization of the bicarbonate-sensing soluble adenylyl cyclase from a bony fish, the rainbow trout Oncorhynchus mykiss. *Interface Focus* **11**, 20200026 (2021).

39. H. L. Li, S. Go, J.-C. Chang, A. Verhoeven, R. O. Elferink, Soluble adenylyl cyclase, the cell-autonomous member of the family. *Biochimica et Biophysica Acta (BBA) - Molecular Basis of Disease* **1870**, 166936 (2024).

40. D. Hahn, J. Tusell, S. Sprang, X. Chu, Catalytic Mechanism of Mammalian Adenylyl Cyclase: A Computational Investigation. *Biochemistry* **54**, 6252–6262 (2015).

41. Q96PN6 - ADCY10, human sAC. *UniProt* (2025). Available at: https://www.uniprot.org/uniprotkb/Q96PN6/entry [Accessed 16 September 2025].

42. O76074 - PDE5A, human. *UniProt* (2025). Available at: https://www.uniprot.org/uniprotkb/O76074/entry [Accessed 16 September 2025].

43. F8RL02 - GC, Arbacia punctulata. *UniProt* (2025). Available at: https://www.uniprot.org/uniprotkb/F8RL02/entry [Accessed 16 September 2025].

44. D. Willoughby, N. Masada, A. J. Crossthwaite, A. Ciruela, D. M. F. Cooper, Localized Na+/H+ Exchanger 1 Expression Protects Ca2+-regulated Adenylyl Cyclases from Changes in Intracellular pH*. *Journal of Biological Chemistry* **280**, 30864–30872 (2005).

45. T. Nishigaki, *et al.*, Intracellular pH in Sperm Physiology. *Biochem Biophys Res Commun* **450**, 1149–1158 (2014).
